# Mechanical Stretch‐Induced Interlayer Coordination between MMP2 and COL17A1 Exacerbates Regenerative Exhaustion in Skin

**DOI:** 10.1002/advs.202511474

**Published:** 2025-09-17

**Authors:** Yidan Sun, Qili Qian, Luwen Xu, Bowen Gao, Ting Li, Yin Li, Jiayi Zheng, Qiaoyu Fu, Xi Cheng, Nuo Chen, Sijia Wang, Liang Zhang, Caiyue Liu, Qingfeng Li

**Affiliations:** ^1^ Department of Plastic and Reconstructive Surgery Shanghai Ninth People's Hospital Shanghai Jiao Tong University School of Medicine Shanghai 200011 China; ^2^ Shanghai Institute of Nutrition and Health University of Chinese Academy of Sciences Chinese Academy of Sciences Shanghai 200030 China; ^3^ Shanghai Key Laboratory of Reproductive Medicine Shanghai 200025 China

**Keywords:** COL17A1 proteolysis, ECM turnover, marimastat, MMP2, regenerative exhaustion

## Abstract

The layered structure of skin necessitates highly sophisticated tissue coordination during regeneration. The unmet clinical need of long‐term skin expansion therapy stems from limited regenerative capacity, yet the underlying mechanism remains enigmatic due to the lack of appropriate animal model. A mouse scalp‐based mechanical stretch model is established that mimics clinical long‐term skin expansion. Prolonged skin expansion progressively drives interfollicular epidermal stem cells towards a state of irreversible regenerative exhaustion, marked by impaired proliferation, differentiation, adhesion, and activity. Mechanistically, mechano‐stress‐induced accumulation of MMP2 in the dermis mediates a shift in extracellular matrix turnover from deposition to degradation, impairing stem cell activity, disrupting niche integrity, and simultaneously triggering proteolysis of COL17A1 at the interlayer. Restoring COL17A1, either through genetic overexpression or administration of Marimastat, a protease inhibitor, is sufficient to mitigate regenerative exhaustion. Consistently, in patient‐derived skin samples, COL17A1 levels correlate with ECM integrity and regenerative potential. Combined, a new stretch‐induced skin expansion model is established, revealing hidden components underlying regenerative exhaustion, and proposing Marimastat for drug repurposing. Restoration of COL17A1 is proposed to provide clinical benefits for skin expansion therapy.

## Graphical abstract

Regeneration reaches a hidden limit under mechanical stretch. Prolonged tension alters dermal–epidermal communication, exhausting epidermal stem cells via MMP2–COL17A1 signaling. MMP2 inhibition by Marimastat preserves adhesion and regenerative capacity, offering a potential strategy to overcome the inherent boundaries of skin expansion.

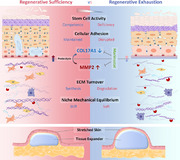

## 1 Introduction

Achieving controlled in situ regeneration of solid organs remains a major clinical challenge.^[^ ^1^ ^]^ Tissue expansion therapy, a first‐line treatment for skin defects,^[^ ^2^ ^]^ leverages the skin's mechanically responsive regenerative capacity through controlled overstretching to stimulate growth and generate new skin. This approach is one of the few clinically available methods for in situ regenerative of human solid organs.^[^ ^3^ ^]^ However, during long‐term expansion (LTE), the skin's regenerative ability to endure persistent mechanical stimulation weakens, resulting in complications such as necrosis, rupture, and breakdown due to regenerative exhaustion (RE).^[^ ^4^, ^5^, ^6^, ^7^, ^8^ ^]^ These issues not only limit the therapy's success and obstruct autologous skin grafting,^[^ ^9^, ^10^ ^]^ but also make LTE a valuable model for studying the mechanisms of RE in mechanically driven skin regeneration.^[^ ^11^, ^12^, ^13^ ^]^ Furthermore, developing strategies to delay regenerative failure is a priority in regenerative medicine, particularly for plastic surgeons worldwide.^[^ ^14^, ^15^ ^]^

Coordinating full‐thickness skin regeneration remains one of regenerative medicine's puzzles, as it requires orchestrating repair of distinct layers while preserving the skin's delicate mechanical property.^[^ ^16^, ^17^, ^18^, ^19^ ^]^ This complexity arises because the processes regulating regeneration in the stem cell‐rich epithelium, the intervening basement membrane (BM), and the stromal‐rich dermis are distinct and asynchronous, each governed by its own lineage‐specific characteristics.^[^ ^17^, ^20^ ^]^ As a stromal‐poor structure, the tightly knit epidermis is anchored to the BM via hemidesmosomes (HDs) and integrins, while cells within this layer interact via desmosomes, adherens junctions, gap junctions, and tight junctions.^[^ ^21^ ^]^ These structural features enable epidermal stem cells to adapt to mechanical cues or injury by adjusting their proliferation, differentiation, and movement.^[^ ^22^ ^]^ In contrast, the stem cell‐poor dermal fibroblasts, which are less regenerative, maintain tissue homeostasis primarily by extracellular matrix (ECM) remodeling and turnover, involving the deposition, rearrangement, or degradation of collagen and elastic fibers to preserve stromal structure and function.^[^ ^23^, ^24^ ^]^ The dermal niche, which provides epithelial scaffold and mechanical resistance, contribute to stem cell behavior and tissue deformation.^[^ ^17^, ^25^, ^26^ ^]^ Insufficient regeneration resulting from impaired mechanical homeostasis contribute to issues like skin aging,^[^ ^27^, ^28^, ^29^ ^]^ chronic non‐healing wounds,^[^ ^30^ ^]^ and skin diseases^[^ ^31^ ^]^ conditions often tied to dysregulation and exhaustion of epithelial stem cells caused by alterations in dermal microenvironment.^[^ ^32^ ^]^ Investigating the regenerative process of mechanically stretched skin provides valuable insight into how interactions between different skin components impact the overall regenerative process.^[^ ^33^ ^]^

Short‐term regenerative responses to mechanical stretching have been extensively studied in vivo,^[^ ^34^ ^]^ revealing increased epithelial stem‐cell renewal, differentiation and the emergence of a “stretch‐responsive” cluster. These processes are driven by the activation of key genes related to cell–cell adhesion, the actomyosin cytoskeleton, and signaling pathways such as EGF‐MAPK‐ERK and YAP‐TEAD. Despite these regenerative effects in epidermis, no neogenesis was observed in hair follicles (HFs), and the dermis showed minimal proliferation.^[^ ^35^ ^]^ Instead, the dermal compartment underwent a pro‐fibrotic transition of myofibroblasts^[^ ^36^ ^]^ and recruitment of immune cells,^[^ ^37^ ^]^ suggesting layer‐specific regenerative responses. While these findings highlight distinct behaviors across skin layers under short‐term expansion, a comprehensive understanding of how long‐term tissue expansion (LTE) influences both epidermal and dermal compartments remains lacking. One major limitation is the scarcity of physiologically relevant models: most existing studies rely on the dorsal skin of mice and rats,^[^ ^34^, ^35^ ^]^ which exhibits low expansion efficiency due to its loose and redundant nature. This fails to recapitulate the mechanical thresholds and clinical complications encountered in human tissue expansion. Consequently, the mechanisms underlying LTE‐induced regenerative decline remain poorly understood.

In the present study, we developed a reliable mouse skin expansion model under the scalp of C57BL/6J mice to facilitate a detailed definition of LTE process. This model allowed us to precisely assess the dynamic transition in epidermal regenerative capacity from sufficient to insufficient. By leveraging this model, we generated a comprehensive single‐cell transcriptomic atlas that elucidates the mechanisms underlying RE. It turns out that stretching induces an imbalance in collagen degradation, driven by MMP2 secretion, which compromises BM integrity, causes COL17A1 proteolysis, and triggers this irreversible exhausted state. Treatment with the MMP inhibitor Marimastat mitigated dermal collagen breakdown and alleviated stretch‐mediated RE. In clinical settings, COL17A1 protein levels also serve as a reliable indicator of ECM turnover balance and IFESCs regenerative potential. These insights advance our understanding of the temporal and spatial dynamics of stretch‐mediated skin regeneration, highlight the importance of ECM mechanical properties in maintaining BM integrity during epidermal regeneration, and propose Marimastat as a promising therapeutic candidate for managing RE in the future.

## 2 Results

### 2.1 Long‐Term Tissue Expansion Leads to Exhaustion of Epidermal Regenerative Capacity

**Figure 1:**
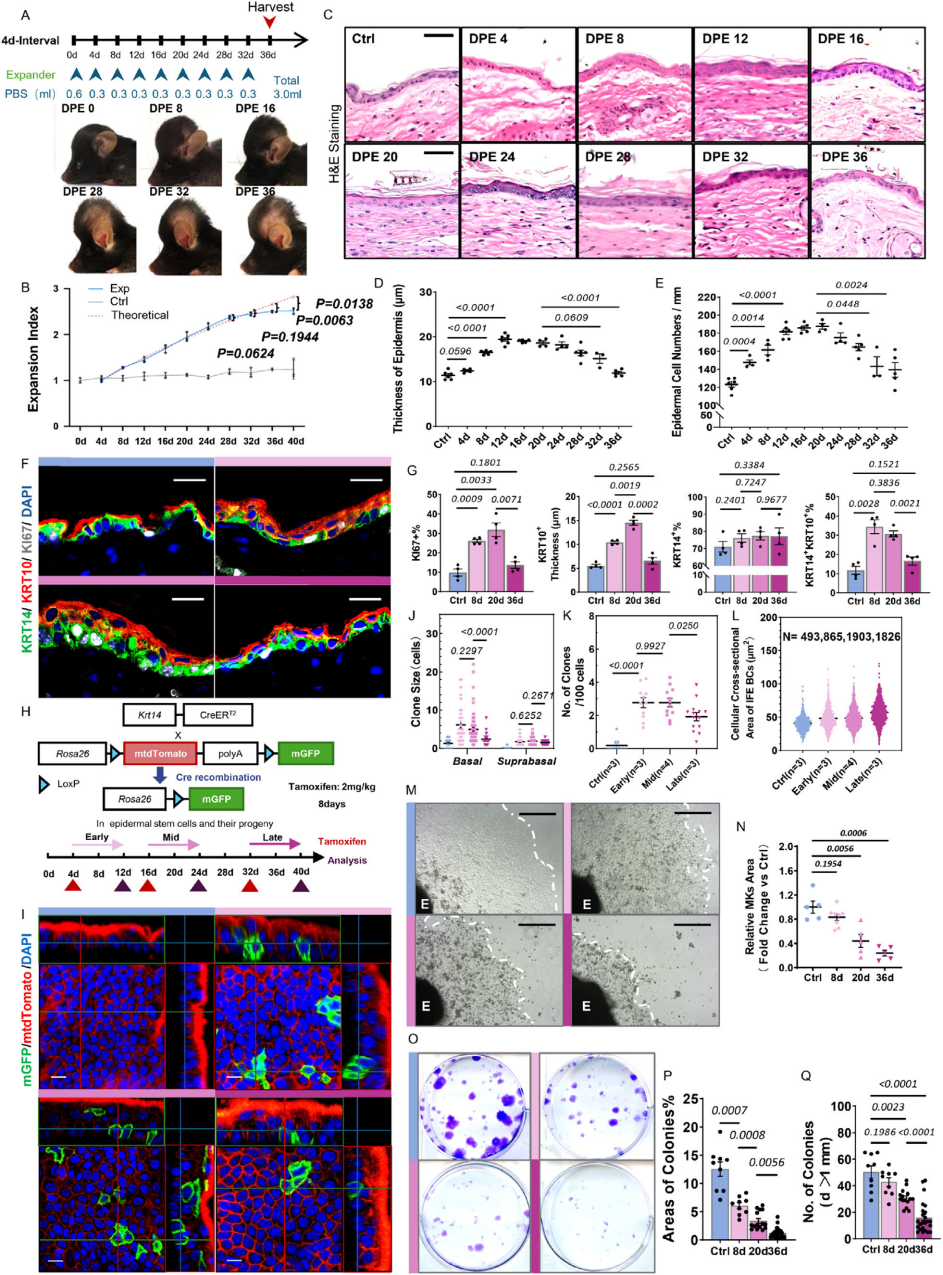
LTE leads to exhaustion of epidermal regenerative capacity. A) Schematic of the 4‐day interval expansion protocol using a mouse scalp model. Representative images show macroscopic skin expansion across indicated time points. B) Expansion index curve comparing experimental vs theoretical expansion, with days post‐expansion (DPE) on the x‐axis. Data are mean ± SEM, *n* = 3 per group. *p*‐values determined using two‐tailed unpaired Student's *t*‐test. C) Representative H&E staining of the epidermis at each time point. Scale bar = 50 µm. D,E) Quantification of epidermal thickness (D) and basal layer cell number per 1 mm basement membrane (E). Data points represent mean values derived from three randomly selected microscopic fields per individual mouse. All time‐course experiments included ≥ 3 biological replicates per time point. F) IF staining of KI67 (white), KRT10 (red), KRT14 (green), and DAPI (blue) showing changes in proliferation and differentiation markers. Yellow regions represent KRT14⁺/KRT10⁺ transitional cells. Scale bar = 20 µm. G) Quantification of KI67^+^, KRT10^+^, KRT14^+^, and KRT14^+^/KRT10^+^ cells per time point. *n* > 3 mice values represent averages from ≥ 3 fields/mouse. H) Schematic of lineage tracing strategy using *Krt14‐CreER; Rosa26‐mTmG* mice. I) Whole‐mount epidermal images of mGFP‐labeled basal cell progeny at indicated time points. Scale bar = 20 µm. J–L) Quantification of clone size (J), clone number per 100 basal cells (K), and cross‐sectional area of basal cells (L). Images were captured at 63 × magnification using a Zeiss confocal microscope. *n =* individual mice; *N =* total number of cells analyzed using Cellpose. M,N) Explant culture showing keratinocyte outgrowth capacity after in vivo expansion. Outgrowth area was measured 4‐days post‐initiation. *n* ≥ 3 biological replicates per group. E, explant. Scale bar = 500 µm. O) Colony‐forming assay of expanded epidermal keratinocytes at indicated times. P,Q) Quantification of IFESC attachment (P) and colony numbers (Q)*. n* = 3 mice per group.

In this study, we developed a murine scalp expansion model using saline injection at 4‐day intervals to investigate the dynamics of skin regeneration under progressive mechanical stretch (**Figure** **1**A). Given that the number of hair follicles (HFs) remains unchanged within a fixed skin area, HF density serves as a spatially stable marker of local skin expansion.^[^ ^35^ ^]^ To quantify expansion efficiency, we introduced the Expansion Index, calculated as the inverse of HF density, which reflects the extent of skin area growth (Figure S1A, Supporting Information). Interestingly, the empirical expansion curve (blue) closely followed the theoretical prediction (red) up to 32 days post‐expansion (DPE 32), suggesting that regeneration capacity kept pace with mechanical expansion during this period. However, beyond DPE 32, the expansion curve plateaued and deviated from the theoretical trajectory, marking a decoupling between expansion volume and actual skin surface area enlargement (Figure 1B). This inflection point signifies the onset of RE, where the skin overlying the expander apex could no longer sustain area growth independently and required lateral tissue supplementation to accommodate further expansion.

Histological analysis revealed a rapid increase in epidermal thickness and cell density during the early phase (DPE 0–12), reflective of successful tissue formation. Upon prolonged treatment (DPE 12–32), thickness and cell density plateaued, indicating that basal stem cells' renewal and differentiation barely kept pace with surface area growth. At the late stage (DPE 36), both thickness and cell density had substantially decreased, consistent with the decline in the expansion index, suggesting diminished regenerative capacity (Figure 1C–E).

Given the pivotal role of basal stem cells in skin self‐renewal, we assessed the dynamics of stem cell populations, including the percentage of proliferative cells (KI67^+^), undifferentiated basal cells (KRT14^+^/KRT10^−^), differentiating cells (KRT14^+^/KRT10^+^), and the thickness of the fully differentiated cells layers (KRT14^−^/KRT10^+^). At DPE36, proliferation (KI67^+^) and differentiation (KRT14^+^/KRT10^+^ and KRT14^−^/KRT10^+^) were significantly dropped, while the basal cell pool (KRT14^+^%/KRT10^−^) remained stable, signifying a waning proliferation and differentiation capacity without effecting stem cell numbers of the IFESCs (Figure 1F,G). Additionally, we observed an increase in KRT10^+^ cells in the basal layer, a phenotype characteristic of aged mouse skin at DPE36 (Figure S1B, Supporting Information).^[^ ^27^ ^]^ Analysis of YAP1, a mechanosensitive factor,^[^ ^38^ ^]^ revealed its nuclear localization (activated) peaked at DPE8, but gradually declined by DPE36 (Figure S1C, Supporting Information).

To assess stem cell dynamics under LTE, we performed single‐cell clonal tracing in *Krt14‐creER* *;* *Rosa* *26* ^*m* *T* *m* *g*^ mice following low‐dose tamoxifen induction (Figure 1H). The distribution and size of labeled clones over a defined tracing period served as indicators of stem cell growth behavior. During the early and mid‐phase, basal clones expanded nearly fourfold compared to the homeostatic control skin. In contrast, both clone size and frequency markedly decreased in the late phase, suggesting that LTE progressively impaired the ability of basal cells to form large clones and limited labeling distribution (Figure 1I–K; Figure S1D, Supporting Information). Consistent with these findings, an ex vivo wound healing assay using skin explants revealed an impairment in keratinocyte outgrowth (Figure 1M). An in vitro self‐renewal assay evaluating regenerative potential, further confirmed the compromised clonogenicity and weakened cell adhesion after repeated expansions (Figure 1M).

Furthermore, measurements of the cross‐sectional area of IFE basal stem cells‐visualized by tdTomato‐labeled cell membranes‐revealed a significant increase in cell size during the late stage. In contrast, cell area remained relatively stable between the early and middle stages (control: 40.71; early: 48.33; middle: 48.61; and late: 57.14 µm^2^) (Figure S1E, Supporting Information; Figure 1L). These findings suggest that IFESCs primarily adapted to early and mid‐stages mainly through increased proliferation, whereas late‐stage cells compensated by cellular enlarging.

To further assess whether RE is primarily driven by cumulative expansion duration or strain magnitude, we compared two protocols that achieved the same final volume but differed in expansion frequency (every 2 vs 4 days) (Figure S1G, Supporting Information). While peak tension was comparable, the accelerated protocol led to more frequent tissue rupture and lower preservation rates, likely due to insufficient adaptation time and uneven stress distribution causing localized necrosis (Figure S1H, Supporting Information). Although skin expanded over shorter durations showed slightly less severe exhaustion phenotypes, key indicators like proliferation, differentiation, mechanosensitive responses, and aging‐related phenotypes compared to longer expansion (DPE36/18), had already begun to decline (Figure S1I, Supporting Information). These results suggest that RE is influenced by both duration and the rate of mechanical loading, underscoring the need to balance mechanical input with biological adaptability in clinical settings.

In summary, while epithelial cells phenotypically adapt to maintain homeostasis during continuous mechanical expansion, their regenerative capacity is gradually compromised and ultimately unable to meet growth demands. This results in impaired proliferation, disrupted differentiation, cellular flattening, and diminished stem cell activity–hallmarks of regenerative exhaustion (RE) caused by prolonged expansion.

### 2.2 LTE Triggers Loss of Proliferation, Differentiation and Stemness in Basal Cells of Interfollicular Epidermis

**Figure 2:**
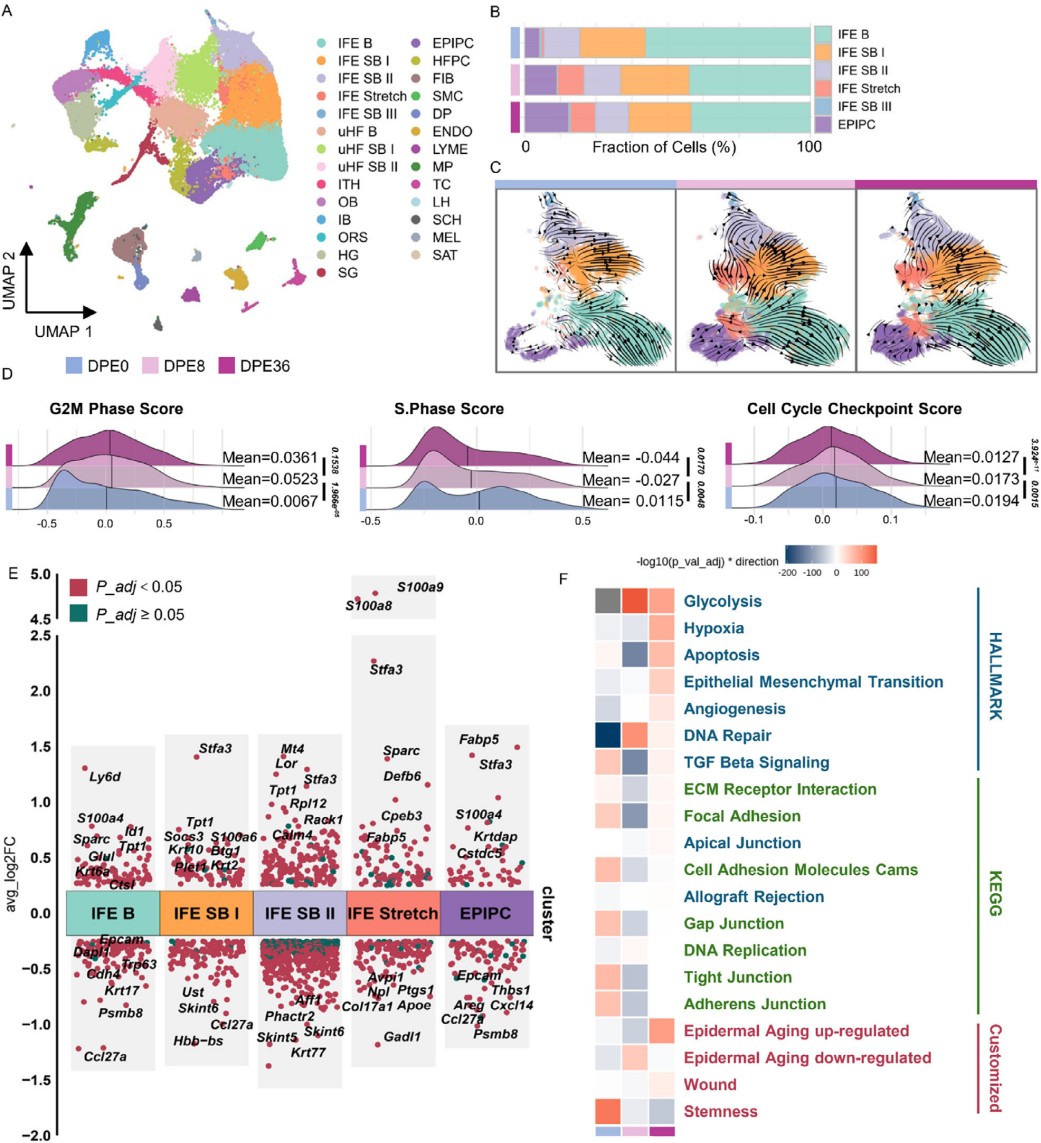
LTE triggers loss of proliferation, differentiation and stemness in basal cells of interfollicular epidermis. A) UMAP plot of scRNA‐seq data from control, DPE8, and DPE36 samples, identifying 27 major cell clusters. Color‐coded and labeled by general identity (right). Abbreviations: IFE B, IFE basal; IFE SB, IFE suprabasal; uHF B, upper hair follicle basal; ITH, isthmus; OB, outer bulge; IB, inner bulge; ORS, outer root sheath; HG, hair germ; SG, sebaceous gland; PC, proliferating cells; FIB, fibroblasts; SMC, smooth muscle cells; DP, dermal papilla; ENDO, endothelial cells; LYME, lymph vessel cells; MP, macrophages; TC, T cells; LH, Langerhans cells; SCH, Schwann cells; MEL, melanocytes; SAT, subcutaneous adipose tissue. B) Proportional distribution of interfollicular epidermal (IFE) cell subtypes across groups. All colors match the clusters in (A) unless noted otherwise. C) RNA velocity projection within IFE compartments reveals altered differentiation trajectories during LTE. Velocity streamlines show the gene‐averaged flow. D) Ridge plots showing reduced enrichment of G2/M phase, S phase, and cell cycle checkpoint gene signatures in proliferative cells (PCs) over time. Mean values and Wilcoxon test *p*‐values are indicated. E) Differential gene expression analysis showing up‐ and down‐regulated genes across IFE clusters (DPE36 vs DPE8). Markers identified using findmarker function, with thresholds: avg_log2FC ≥ 0.25 & pct.1 ≥ 0.25 & pct.2 ≥ 0.25. Adjusted *p*‐values < 0.05 in red, ≥ 0.05 in dark green. F) Heatmap of selected pathway activities across time points. Color scale (blue to red) represents −log10(adjusted *p*‐value) × direction of change.

To elucidate how LTE impacts IFESCs, we analyzed single‐cell RNA transcriptomics from isolated skin cells upon short‐ (8 days) and long‐term (36 days) skin expansion, as well as from non‐expanded controls (49‐day‐old postnatal mice). After quality control (including doublet removal, batch effect correction, and normalization), we obtained gene expression profiles of 61687 cells across 27 distinct clusters (**Figure** **2**A; Table S1, Supporting Information).

In line with previous findings,^[^ ^34^ ^]^ we identified a unique “IFE_Stretch” subpopulation arising from interfollicular epidermis in stretched skin (Figure 2A,B). This cluster featured elevated expressions of basal (*Krt14, Itga6, Itgb1*), hyperproliferation (*Krt6a, Krt6b, Krt16*) and inflammation markers (*S100a8, S100a9*) (Figure S2A, Supporting Information). Lineage trajectory analysis via scVelo revealed dominant epithelial trajectories, progressing from basal stem cells (IFE B) to spinous (IFE SB I) and granular cells (IFE SB II and III). IFE_Stretch cells emerged as an intermediate transitioning population between IFE_B and IFE_SB II, particularly enriched in stretched samples. However, this transition became impaired at DPE36, as reflected by reduced RNA velocity vectors, indicating diminished differentiation potential after prolonged expansion (Figure 2C).

To examine how LTE affects cell proliferation, we inferred cell cycle states across proliferating cells (PCs). At DPE36, PCs exhibited reduced G2/M and S‐phase scores and weakened checkpoint signals (Figure 2D, Figure S2B, Supporting Information). Heterogeneity analysis further subdivided PCs into four IFE_basal subtypes (PC_ IFEB 1–4), one IFE_stretch, three HF‐associated (PC_uHF B, PC_OB, PC_ HG) and one immune subpopulation (Figure S2C, Supporting Information). This analysis demonstrated that both IFE and HF, but not fibroblasts, contributed to in situ skin proliferation (Figure S2C,D, Supporting Information). Among them, PC_IFEB_4, characterized by high expression of *Thbs2, Pcna, Epgn*–genes related to nuclear function and DNA replication—was significantly reduced in the DPE36 group (*p* = 0.04885), suggesting impaired DNA replication and delayed cell cycle progression (Figure S2D,E, Supporting Information).

Next, differential expression analysis of basal IFE cells (IFE_B) between DPE36 and DPE8 revealed that *Krt6a* (a wound healing marker gene) and *S100a4* (an epithelial‐mesenchymal transition, EMT marker gene) were upregulated at DPE36, along with downregulation of *Trp63* (an epithelial stemness marker gene)*, Epcam* (Epithelial Cell Adhesion Molecule), and *Cdh4* (calcium‐dependent cell adhesion gene) in the late stage. Additionally, genes encoding cysteine endopeptidase inhibitors (*Stfa3, Cstdc5*)^[^ ^30^ ^]^ were broadly upregulated, while the chemokine *Ccl27a*, important for lymphocyte recruitment by keratinocytes,^[^ ^39^ ^]^ was markedly suppressed (Figure 2E).

To capture global pathway changes, we performed GSEA using the AUCell method (irGSEA package).^[^ ^40^ ^]^ At the late stage (DPE36), there was enrichment of hypoxia, apoptosis, EMT, and angiogenesis pathways. Meanwhile, cell adhesion‐related pathways—ECM‐receptor interaction, focal adhesion, tight junctions, gap junctions, and adherens junctions—were consistently downregulated. In contrast, glycolysis and DNA repair pathways were predominantly activated at the early stage (DPE8). Further comparative analysis using customized gene sets from aged epidermis^[^ ^41^ ^]^ (GSE133648), wound response^[^ ^42^ ^]^ (GSE142471) and stemness^[^ ^43^ ^]^ (GSE70523) revealed that basal stem cells upon LTE demonstrated features of gene expression observed in aging, would response, as well as impaired stemness (Figure 2F). Inferred cell‐cell communication analysis using CellChat^[^ ^44^ ^]^ revealed that fibroblast‐derived basement membrane components – including collagen IV (*Col4*), collagen VI (*Col6*), and various laminin isoforms^[^ ^45^, ^46^, ^47^ ^]^–consistently interacted with basal IFE mechanosensitive receptors such as integrins (e.g., *Itga2* */Itgb1*)^[^ ^48^ ^]^ and *CD44* ^[^ ^49^ ^]^ across different stages of expansion. Notably, as expansion progressed, laminin–integrin signaling from IFE B to fibroblasts became more prominent, suggesting a shift in reciprocal niche communication (Figure S2F, Supporting Information).

### 2.3 Delayed Hair Cycle Response after LTE

Mammalian HFs play a crucial role in skin regeneration under various conditions and are housed within specialized niches in the skin.^[^ ^50^, ^51^, ^52^ ^]^ As for the long‐term stretch condition, we identified a subpopulation corresponding to outer root sheath (ORS) cells, specifically present in DPE36 samples (Figure S2G, Supporting Information). This subpopulation exhibited high expression of anagen HF marker genes,^[^ ^53^ ^]^ including *Gja1*, *Krt17*, *Barx2*, and *Krt6a*. We also observed an increased proportion of uHF SB II population, marked by high expression of *Cst6*, *Defb6*, and *Klk10* (Table S1, Supporting Information) along with a reduction in outer bulge (OB) cells. Further DEG analysis revealed a downregulation of typical telogen bulge markers like *Cd34* ^[^ ^54^ ^]^ in the OB (Figure S2H, Supporting Information).

Morphological examination of contemporaneous control skin samples, collected at 80‐days postnatal, confirmed that HFs had entered a fully developed anagen phase, displaying typical anagen HF structures, whereas HFs collected at DPE36 remained in the telogen phase (Figure S2I, Supporting Information). IF analysis demonstrated the absence of ectopic *Krt10* expression^[^ ^55^ ^]^ in long‐term expanded bulge hair follicle stem cells (HFSCs), while confirming the presence of proliferating cells in the upper hair follicle (uHF), hair germ (HG), and sebaceous glands (SGs) (Figure S2J, Supporting Information). We also found SGs gradually enlarged following serial stretching (Figure S2K,L, Supporting Information). In conclusion, we preliminary confirmed that mechanical stretching delays HF morphogenesis and subsequent hair cycling, while also leading to the enlargement of SGs.

### 2.4 LTE Shifts Collagen Turnover Towards Degradation and Promotes MMP2 Accumulation in Dermis

Stem cell fate decisions are governed by both intrinsic factors and the surrounding niche microenvironment.^[^ ^56^ ^]^ Beneath the epidermis, the dermis houses mesenchymal cells,^[^ ^57^, ^58^ ^]^ which modulate stem cell behavior through ECM turnover and remodeling or by secreting growth factors.^[^ ^59^ ^]^ To further understand LTE affects the IFESC niche, we next focused on dermal alterations.

**Figure 3:**
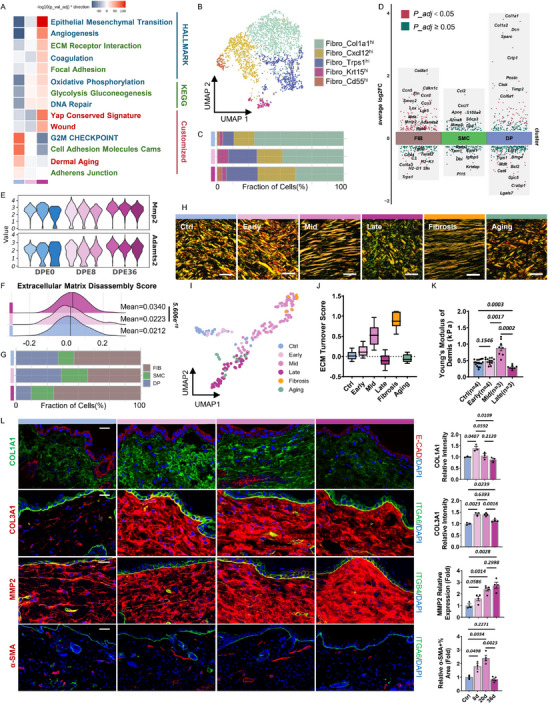
LTE shifts collagen turnover towards degradation and promotes MMP2 accumulation in dermis. A) Heatmap of representative gene set enrichment scores between groups using irGSEA package in fibroblasts. Color keys (blue to red) reflect the range of −log10(*p* adj value)^*^ direction. B) UMAP plot depicting subcellular heterogeneity of fibroblasts (FIBs). C) Bar plot illustrating the fraction of FIB subclusters in each group. D) Differential gene expression analysis showing up‐ and down‐regulated genes across mesenchymal‐lineage clusters between DPE36 and DPE8. E) Ridge plots displaying ECM disassembly score enrichment in FIBs. F) Violin plots for representative genes (*Mmp2, Adamats2*) expression in FIBs. G) Bar plot illustrating the fraction of mesenchymal‐lineage clusters in each group. H) Representative birefringence images of picrosirius red‐stained dermal sections from control, expanded, wound‐induced fibrotic, and aged skin. I) Combined UMAP visualization of ECM ultrastructural features in control skin, skin expanded for varying durations, wound‐induced fibrotic skin, and aged skin. For expansion conditions, analysis was performed on over six regions from at least five individuals per time point. J) A machine learning‐based ECM turnover score was generated from picrosirius red‐stained dermal images, ranging from 0 (homeostatic state) to +1 (increased collagen deposition). Negative values indicate net collagen degradation. K) Stiffness of the dermis measured by AFM, represented as Young's Modulus (kPa). Data points indicate mean values derived from multiple stiffness curves per views (*n* ≥ 3 biological replicates per group). L) IF staining and quantification of the relative fold change in IF intensity of COL1A1 (green), COL3A1 (red), MMP2 (red), α‐SMA (red), and DAPI (blue) in skin sections at indicated time points. Mean value of IF intensities from control samples as a standard value unless otherwise noted. Scale bar = 20 µm.

Histological analysis revealed minimal dermal proliferation and a gradual reduction in dermal thickness, failing to match the rate of epidermal growth (Figure S3A,B, Supporting Information). GSEA of fibroblasts showed activation of stretch‐induced pathways, including EMT, ECM‐receptor interaction, and focal adhesion, while signatures related to cell adhesion molecules (CAMs), adherens junctions, and the G2/M checkpoint were suppressed at DPE36 (Figure **3**A). Additionally, metabolic pathways, including angiogenesis, coagulation, oxidative phosphorylation, and glycolysis/gluconeogenesis, were upregulated at DPE36. A DNA repair response was notably triggered at DPE8, indicating a rapid chromatin mechanoresponse. Custom gene set analysis further demonstrated significant enrichment of YAP activation^[^ ^60^ ^]^ and wound healing^[^ ^61^ ^]^ (GSE188432) at DPE36, while dermal aging‐related signatures^[^ ^62^ ^]^ (CRA004660) remained unchanged, indicating that LTE does not activate aging‐related programs (Figure 3A). Further GO enrichment at DPE36 revealed upregulated processes in ECM remodeling, wound healing, cell motility, TGF‐β signaling, and integrin binding, while downregulated terms were primarily linked to transcription inhibition (Figure S3C, Supporting Information).

The dynamic balance of ECM turnover relies on the equilibrium between collagen synthesis and degradation.^[^ ^23^, ^26^, ^63^ ^]^ We first assessed fibroblast heterogeneity and identified five distinct subpopulations with unique molecular signatures (Figure 3B; Figure S3D, Table S2, Supporting Information): *Col1a1^high^, Cxcl12^high^, Trps1^high^, Krt15^high^, and Cd55^high^*. Notably, *Col1a1^high^* fibroblast subset, critical for ECM organization, showed a progressive decline in proportion by DPE36, suggesting compromising dermal structural integrity and support (Figure 3C; Figure S3D, Supporting Information). Next, comparing DEGs in fibroblasts between DPE36 and DPE8, we observed upregulation of ECM‐degrading proteinase‐*Mmp2* and *Adamts2*‐while most other proteinases remain unchanged (Figures 3D,E; Figure S3E, Supporting Information). Consistently, ECM disassembly pathway was specifically enriched at the late stage (Figure 3F), highlighting active collagen degradation. In contrast, smooth muscle cells (SMCs), typically contractile myofibroblasts contributing to collagen synthesis,^[^ ^58^ ^]^ did not increase at DPE36 as they did at DPE8 (Figure 3G), suggesting an impaired fibroblast‐to‐myofibroblast transition and reduced ECM synthesis. Together, these results indicate that prolonged mechanical expansion disrupts collagen turnover homeostasis by favoring degradation over synthesis, thereby weakening dermal ECM structure and mechanical integrity and epidermal stem cell support.

To further explore ECM regulatory, we examined several key transcripts. Fibrosis‐related genes such as *Col8a1*,^[^ ^64^ ^]^ *Lgr5* (scleroderma‐related)^[^ ^65^ ^]^ were upregulated, along with *Ccn5* (anti‐fibrotic^[^ ^66^ ^]^), *Loxl* (collagen crosslinking^[^ ^67^ ^]^), *Eln* (elastic fiber synthesis^[^ ^68^ ^]^) and *Cdkn1c* (proliferation inhibitor^[^ ^69^ ^]^) were upregulated; while dermal regeneration‐associated gene *Trps1* ^[^ ^70^ ^]^ was downregulated (Figure 3D).

To investigate ECM structural changes, we performed picrosirius red staining combined with a machine learning‐based algorithm^[^ ^70^, ^71^ ^]^ to quantitatively assess ECM fiber ultrastructure (see Methods). UMAP analysis revealed that during early to mid‐expansion, collagen fibers became longer, thicker, and more aligned along the stretch axis—resembling wound‐induced fibrosis^[^ ^71^ ^]^ and indicative of increased ECM deposition. By DPE36, however, fibers appeared sparser, shorter, and disorganized, with an elevated COL3/COL1 ratio, resembling the fragmentation pattern observed in aging‐related ECM degradation^[^ ^72^, ^73^ ^]^ (Figure 3H,I). To quantify these shifts, we developed an ECM Turnover Score to reflect the balance between collagen deposition and degradation. The average scores were: young control (0.0259); early expansion (0.1354); mid expansion (0.5945); late expansion (−0.09515); stretch‐induced fibrosis (0.8814); and aging‐induced degradation (−0.0451) (Figure 3J). These findings indicate a temporal transition from ECM accumulation to degradation under prolonged expansion, accompanied by altered dermal mechanical properties. Consistent with this, AFM measurements showed that dermal stiffness peaked during mid‐stage ECM deposition but significantly declined at the late stage (Figure 3K), confirming mechanical softening of the dermis under LTE.

IF analysis corroborated these findings: MMP2 protein progressively accumulated during skin expansion, accompanied by degradation of COL1A1 and COL3A1, and a decline in α‐SMA^+^ cells proportion (Figure 3L). Co‐localization of MMP2 with the basement membrane protein ITGB4 suggested its involvement in collagen degradation at the dermal‐epidermal junction (Figure 3L). In contrast, MMP9–a gelatinase homologous to MMP2^[^ ^74^, ^75^ ^]^–was not accumulated in ECM under prolonged expansion (Figure S3F, Supporting Information), suggesting its minimal involvement in ECM remodeling under these expansion conditions. Moreover, nuclear YAP activation was observed in PDGFRα^+^ fibroblasts but not in F4/80^+^ macrophages (Figure S3G, Supporting Information). Fibroblast outgrowth and proliferative capacity were also significantly impaired at DPE36 (Figure S3H,I, Supporting Information).

These results suggest that under LTE, sustained MMP2 activation leads to dysregulated ECM turnover characterized by excessive collagen degradation, reduced dermal stiffness, and impaired niche support. These microenvironmental changes coincide with RE phenotypes and defective IFESC mechanotransduction in the epidermis at DPE36.

### 2.5 LTE‐Induced Regenerative Exhaustion Triggers Irreversible Basement Membrane Disruption and Hemidesmosome Depletion

Transcriptional analysis at DPE36 revealed substantial changes in genes related to cellular adhesion, with a notable enrichment of epithelial‐mesenchymal transition (EMT)‐related genes at the late stage of expansion. Early EMT is now characterized by a dampening of epithelial traits, including compromised cell–cell junctions, loss of apical‐basal polarity, and invasive BM contact.^[^ ^76^, ^77^, ^78^ ^]^ These observations motivated a deeper investigation into the ultrastructural changes occurring in epidermal adhesion during prolonged expansion, using transmission electron microscopy (TEM).

**Figure 4:**
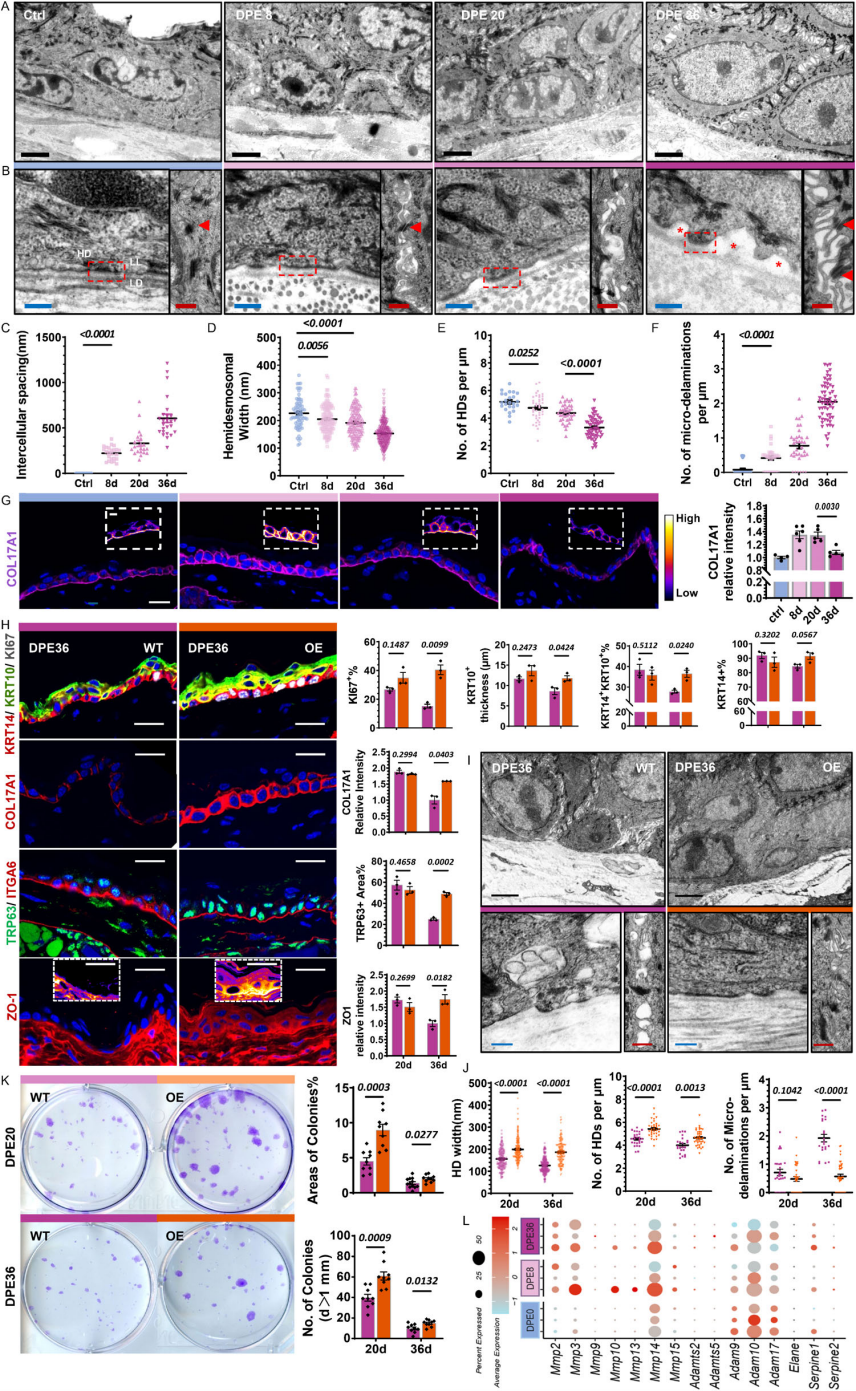
Hemidesmosomes(HDs) Breakdown of IFE Impairs Epidermal Regeneration and Epidermal COL17A1 OE rescues LTE‐RE. A) TEM images of skin tissues from Ctrl and DPE8, DPE20, and DPE36. Scale bar = 2 µm (black). B) Enlarged views of the ultrastructure demonstrate cell‐basement membrane interactions and intercellular adhesions across samples, featuring: HDs (highlighted by red dashed box), lamina lucida (LL), lamina densa (LD), micro‐delamination (*), and desmosomes (marked by red arrows). Filopodia structures are observed between adjacent desmosomes. Scale bars = 200 nm (blue) and 500 nm (red). C–F) Quantified parameters between groups: Intercellular spacing, HD width, HD density (/1 µm BM), and micro‐delaminations (/1 µm BM). G) IF staining of COL17A1 (purple) with quantification of relative intensity. Scale bar = 20 µm. Protein expressions are visualized with a pseudo‐color gradient from black (no expression) to yellow (maximal expression). H) *C* *ol17a1* overexpression (OE) in IFESCs restores proliferation, differentiation, stemness (TRP63), and epidermal tight junction integrity (ZO‐1) in DPE36 after LTE. Scale bar = 20 µm. I,J) TEM ultrastructural analysis shows restoration of cellular adhesion compared from WT and *Col17a1*‐OE mice at DPE36, highlighting HD width, HD density, and micro‐delaminations per 1 µm of BM. K) Colony‐forming assay images of WT and *Col17a1*‐OE expanded epidermal keratinocytes at DPE20 and DPE36, along with quantification of colony growth area and attachment rate. L) Dot plots showing the dynamic expression changes of protease‐related genes involved in COL17A1 post‐transcriptional proteolysis in IFEB cells. The size of each dot indicates the proportion of cells expressing the gene, while the color intensity reflects the average expression level of the gene. All blue nuclei in IF staining images represent DAPI staining unless otherwise specified.

TEM analysis revealed a progressive widening of intercellular spaces between basal cells, accompanied by altered basal stem cell polarity, disrupted tight junction, structurally intact desmosomes and the emergence of filopodia‐like adherens junction structure^[^ ^79^ ^]^ (**Figure** **4**A–C). By DPE36, HD width and density were markedly reduced, while BM micro‐delamination became significantly more frequent, along with disorganized filopodia losing contact with neighboring epithelial cells (Figure 4D–F).

Given that the DPE18 sample from the 2‐day interval expansion protocol maintained relatively intact mechanoresponsiveness and regenerative function (Figure S1G–I, Supporting Information), we next sought to determine which ultrastructural alterations most directly contribute to the onset of RE. We first observed similarly increased intercellular spacing and decreased HD density between DPE10 and DPE18, while HD width remained relatively stable, and BM micro‐delamination showed only a mild increase (Figure S4A,B, Supporting Information). In contrast, the 4‐day protocol showed further decreases in HD width, with substantially higher frequencies of BM delamination (Figure 4C–F; Figure S4A,B, Supporting Information).

These findings suggest that progressive BM micro‐delamination triggered by HD loss is a central event that compromises epidermal integrity and triggers RE. To further elucidate the molecular basis underlying these structural changes, we analyzed the expression of key adhesion‐related proteins, including HD components (COL17A1, ITGA6, ITGB4, and PLECTIN‐1)^[^ ^21^ ^]^ and tight junctions (ZO‐1)^[^ ^78^ ^]^ at various time points. Among these, COL17A1 expression closely mirrored the trend of regenerative potential during LTE (Figure 4G). Moreover, non‐hemidesmosomal COL17A1 expressions increased in the apicolateral regions of basal keratinocytes (Figure 4G). Other HD components displayed distinct expression patterns: ITGA6 exhibited a gradual increase over time; ITGB4 levels specifically declined at DPE36; and PLECTIN‐1evels dropped immediately after stretch (Figure S4C, Supporting Information). Among tight junction proteins, ZO‐1 expression was elevated during early and mid‐stages of expansion but declined at DPE36, further reflecting compromised epithelial barrier integrity.

### 2.6 Irreversible Loss of Epidermal Regeneration Despite Stretch Removal

To evaluate whether LTE‐induced RE represents a reversible adaptation or a persistent impairment, we performed a deflation experiment following 36‐days of expansion. Skin was harvested 8‐days after deflation (Defl) and compared with samples maintained under sustained stretch (Sus) (Figure S5A, Supporting Information). Histological analysis revealed that although dermal thickness recovered in the Defl group, neither epidermal thickness nor basal cell density showed significant restoration (Figure S5B,C, Supporting Information). IF staining showed sustained reductions in basal cell proliferation (Ki67^+^%), differentiation (KRT10^+^ layer thickness), proportion of differentiating cells (KRT14^+^KRT10^+^%), and nuclear YAP localization, indicating persistent loss of mechanotransductive activity and a functionally exhausted epidermal state despite stretch release (Figure S5D,E, Supporting Information). Consistently, clonal growth and cell adhesion capacity of basal keratinocytes could not be restored after deflation (Figure S5F,G, Supporting Information).

TEM examination revealed partial structural recovery, with narrowed intercellular spaces and attenuated BM micro‐delamination. However, HD density remained low and HD width recovered only modestly (Figure S5H,I, Supporting Information). Expression of MMP2, which was previously upregulated under stretch, decreased after deflation, suggesting its role as a tension‐sensitive effector of BM remodeling (Figure S5J,K, Supporting Information).

Together, these findings demonstrate that LTE induces an irreversible state of regenerative exhaustion, marked by sustained depletion of regenerative competence.

### 2.7 Epidermal Over‐Expression of *C* *ol17a1* Rescues LTE‐RE

The stable attachment of basal keratinocytes to BM via HDs is of fundamental importance for maintaining skin integrity.^[^ ^80^, ^81^ ^]^ COL17A1, a primary component of BM and a key marker of interfollicular epidermis (IFE) basal stemness, plays a pivotal role in enhancing the self‐renewal capacity of epidermal stem cells.^[^ ^82^ ^]^ To explore its functional impact, we developed *Krt14‐creERT2; Rosa26‐CAG‐LSL‐Col17a1* mice, enabling targeted overexpression (OE) of *Col17a1* in epidermal stem cells (Figure S4D, Supporting Information).

OE of *C* *ol17a1* did not alter epidermal homeostasis when compared to their wild‐type (WT) littermates (Figure S4E,F, Supporting Information). However, at DPE36, *C* *ol17a1* OE significantly rescued several RE‐related phenotypes, including diminished proliferation, impaired differentiation, compromised stemness, and weakened tight junctions, all of which are characteristic of LTE (Figure 4H). However, at DPE20, when COL17A1 expression remains naturally high in response to stretch, *C* *ol17a1* OE had no additional impact (Figure S4G, Supporting Information). Consistently, ultrastructural analysis of *Col17a1* OE mice revealed longer and denser HDs, reduced micro‐delamination, and more intact BM integrity and epidermal adhesion at late stage (Figure 4I,J), while no significant differences were observed at mid stage when COL17A1 levels remain physiologically elevated (Figure S4H, Supporting Information). *C* *ol17a1* OE did not influence dermal ECM turnover rate (Figure S4I, Supporting Information). Furthermore, expanded keratinocytes in C*ol17a1* OE mice exhibited enhanced clonogenic potential, forming significantly larger colonies and a greater number of colonies than control mice (Figure 4K). This indicates that COL17A1 is critical in preventing RE during LTE and represents a potential target for improving skin expansion.

While mRNA levels of *Col17a1* exhibited only minor changes between DPE8 and DPE36 (Figure S4J, Supporting Information), its protein expression level was significantly reduced at DPE36(Figure 4G). The discrepancy between *Col17a1* mRNA and protein levels suggests potential post‐transcriptional regulation, possibly through proteolytic degradation of COL17A1.^[^ ^83^, ^84^, ^85^ ^]^ Interestingly, further investigation into the mechanisms of COL17A1 proteolysis revealed that the IFEB themselves do not secrete any proteinase capable of mediating COL17A1 post‐transcriptional degradation (Figure 4L). This highlights the need to explore alternative mechanisms responsible for COL17A1 degradation.

### 2.8 MMP2 Mediated Dermal Collagen Degradation and COL17A1 Proteolysis In Vitro

**Figure 5:**
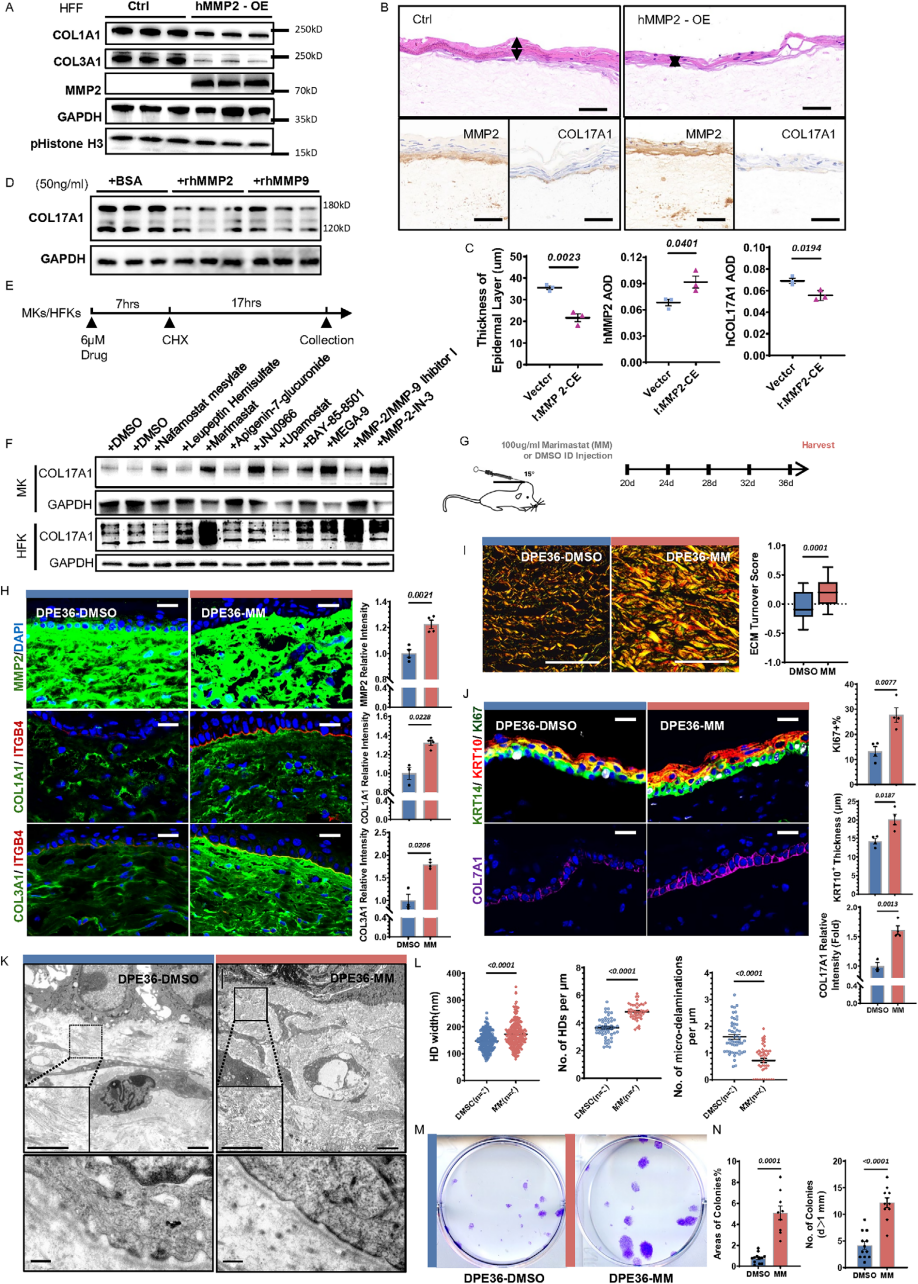
MMP2 mediated dermal collagen degradation and COL17A1 proteolysis in vitro, while Marimastat restores dermal structure and rescue LTE‐RE in vivo. A) Western blots analysis of COL1A1, COL3A1, MMP2, GAPDH and p‐Histone H3 in cultured HFF cells stably transduced with *MMP2* OE or control vector. GAPDH serves as loading control. B) Representative H&E staining and IHC staining of MMP2 and COL17A in 3D human skin equivalents reconstructed with either *MMP2* OE or empty vector HFF cells. Scale bar = 50 µm. C) Quantification of epidermal layer thickness and average optical density (AOD) of MMP2 and COL17A1 staining in the 3D skin equivalents. D)Western blots analysis of COL17A1 in HFK cells treated with recombinant MMP2, MMP9, or BSA (50 ng mL^−1^), showing proteolytic degradation by MMP2. E) Schematic workflow of a drug screening assay in HFKs and MKs pre‐treated with candidate drugs and cycloheximide (CHX), followed by COL17A1 stability assessment. Drug application concentration is set at 6 µM, with 10 µM CHX in HFKs and 3 µM CHX in MKs. F) Western blot results showing COL17A1 levels after drug treatments in MKs and HFKs. Several protease inhibitors (e.g., Marimastat) preserved COL17A1 under CHX‐induced translational blockade. G) In vivo experimental design for intradermal (ID) delivery of Marimastat (MM, *n* = 4) or DMSO control (*n* = 3) from DPE20–36, followed by phenotypic analysis. H) Representative IF images and quantification of MMP2 (green), COL1A1 (green), COL3A1 (green) staining in DPE36 skin tissues treated with MM or DMSO. Scale bar = 20 µm. I) Birefringence imaging of Picrosirius Red‐stained dermis showing collagen architecture and ECM turnover scores in MM‐ and DMSO‐treated groups. Scale bar = 50 µm. J) IF staining and quantification of KI67 (white), KRT10 (red), KRT14 (green) and COL17A1 (purple) in DMSO‐ and MM‐treated mice at DPE36. Scale bar = 20 µm. K,L) TEM showing HD and dermal collagen ultrastructure. HD width, density, and micro‐delamination events quantified. Scale bars: 2 µm (upper), 200 nm (lower). M,N) Colony formation assay of epidermal cells isolated from MM‐ or DMSO‐treated mice, with quantification of colony number and area. Data are presented as mean ± SEM; *p*‐values calculated by unpaired two‐tailed *t*‐tests.

Notably, the collagenolytic activity of MMP not only serves an anti‐fibrotic role in the dermis but also exerts proteolysis effects on adjacent tissues, including BM and intercellular junctions.^[^ ^74^, ^86^, ^87^, ^88^ ^]^ To understand the RE response to mechanical stress, the potential impact of this dermis‐secreted protease on epidermal function and COL17A1 proteolysis warrants close attention. First, *MMP2* OE in HFFs verified the proteolytic effects of MMP2 protein on COL1 and COL3, with a relatively minor effect on fibroblasts proliferation (**Figure** **5**A). Second, primary HFKs treated with recombinant human (rh) MMP2 and MMP9 protein (used as positive control^[^ ^89^ ^]^) showed both the 180‐kD COL17A1 and its shed 120 kD ectodomain were susceptible to MMP2 proteolysis in vitro (Figure 5B), providing evidence of their direct interaction. Moreover, full‐thickness human skin equivalents model was engineered by incorporating HFKs along with either *MMP2* OE or empty vector HFF collagen gel in dermal layer (Figure S6A, Supporting Information). The reconstituted epidermis in the *MMP2* OE group was thinner, showed impaired stratification and reduced COL17A1 protein levels at the interface between the reconstructed keratinocyte and fibroblast layers, compared to the empty vector group (Figure 5C,D). These suggest that dermal MMP2 up‐regulation can lead to COL17A1 degradation and tissue hypotrophy in epidermis. Moreover, collectively, these results indicate that active MMP2 degradation may be the cause for the RE phenotypes observed during LTE.

### 2.9 Marimastat Restores Dermal Structure and Rescue LTE‐RE In Vivo

Given that multiple protease inhibitors have been developed to prevent COL17A1 proteolysis,^[^ ^83^, ^84^ ^]^ we conducted in vitro drug screening using both HFKs and murine keratinocytes (MKs) to identify potential therapeutic agents capable of rescuing LTE‐RE. A panel of 59 protease inhibitors targeting MMPs, serine proteases, and neutrophil elastase (see Table S3, Supporting Information) was initially screened (Figure 5E; Figure S6B, Supporting Information). From this screen, 10 candidates were selected for secondary evaluation based on their ability to prevent COL17A1 degradation in the presence of the protein synthesis inhibitor cycloheximide (CHX) ^[^ ^83^ ^]^ (Figure 5F). Among both human and murine keratinocytes, marimastat—a collagen‐based peptidomimetic and MMP competitive binding inhibitor^[^ ^90^ ^]^—was the most effective in stabilizing COL17A1 protein and prevented its degradation in vitro, without affecting its transcription levels (Figure S6C, Supporting Information).

Intradermal (ID) injections of 100 µg mL^−1^ marimastat or DMSO were administered every other day from DPE20 to DPE36 in the expansion model to assess the in vivo protective effects (Figure 5G). Since Marimastat acts through competitive inhibition rather than suppressing protease synthesis,^[^ ^91^, ^92^ ^]^ a slight upregulation of MMP2 expression was observed; nevertheless, dermal collagen degradation was significantly reduced (Figure 5H), and the ECM turnover score shifted toward collagen deposition (Figure 5I). At the epidermal level, IF analysis revealed enhanced basal cell proliferation, restored differentiation, and preserved COL17A1 expression following Marimastat treatment (Figure 5J). Ultrastructural analysis further showed the reversal of BM disruption, evidenced by increased HD width and density, along with reduced micro‐delamination (Figure 5K,L). Restoration of collagen fiber architecture was also confirmed (Figure 5K). Post‐treatment, the epithelial integrity was supported by elevated expression of ITGB4 and ZO‐1(Figure S6D, Supporting Information). Functionally, colony‐forming assays showed improved clonogenic capacity of IFESCs in vitro after LTE following marimastat administration (Figure 5M).

In summary, these findings highlight Marimastat as a promising therapeutic agent capable of preserving the dermal microenvironment, restoring COL17A1 levels, and protecting epithelial BM integrity, which are critical for maintaining stem cell function and delaying RE after LTE.

### 2.10 Human COL17A1 Protein Levels as Indicators of Regenerative Potential in Skin Expansion Therapy

**Figure 6:**
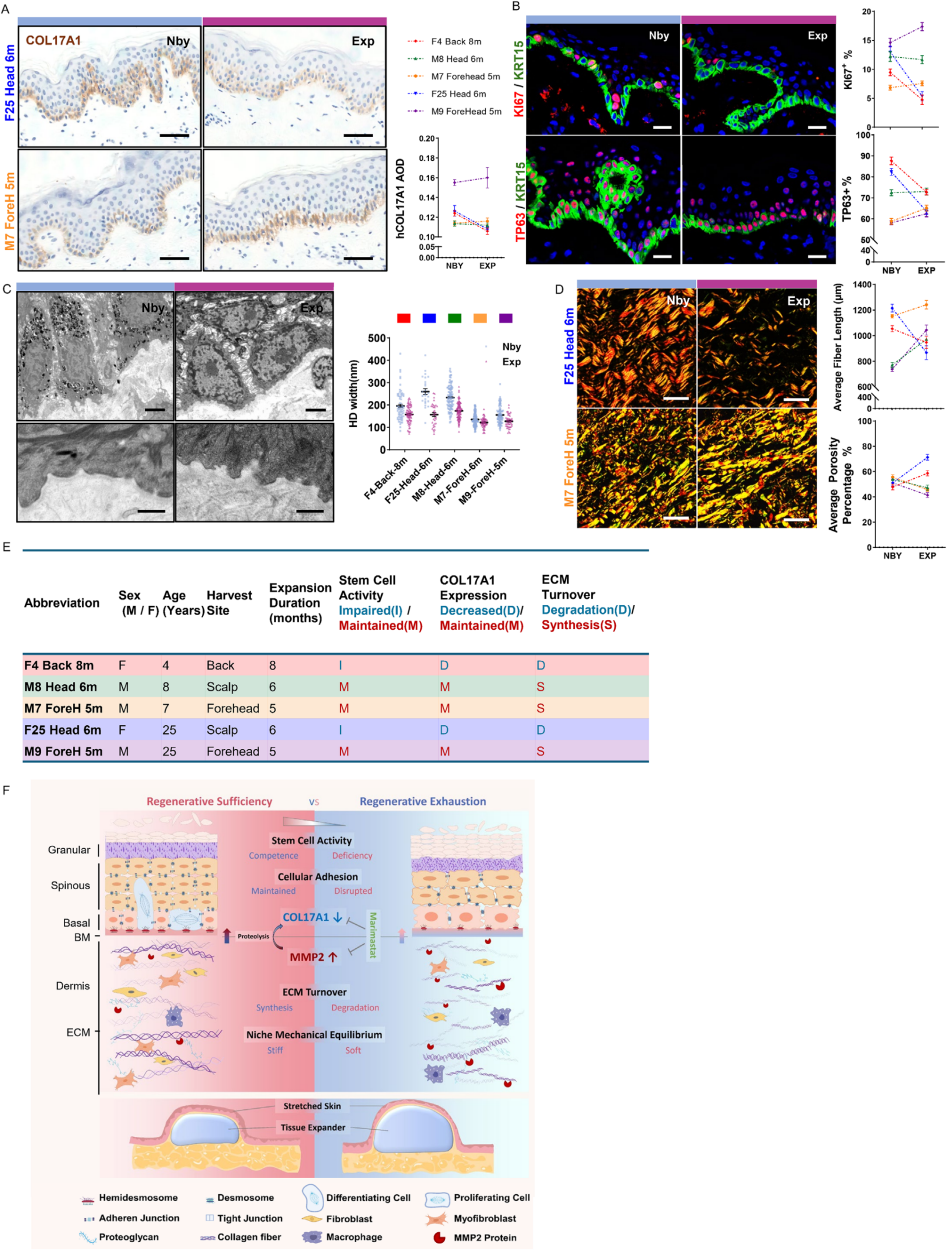
Human COL17A1 Protein Levels as Indicators of Regenerative Potential in Skin Expansion Therapy. A) Representative IHC staining of COL17A1 in nearby (Nby) and expanded (Exp) human skin from different patients. Quantification of average optical density (AOD) is shown (right). Scale bar = 50 µm. B) IF staining of KI67 (red), TP63 (red), KRT15 (green) in Nby and Exp epidermis. Quantification of the percentage of KI67⁺ and TP63⁺ basal cells is shown (right). Scale bar = 20 µm. C) TEM images of HD structure in Nby and Exp human epidermis. Representative ultrastructure shown at low and high magnification. HD width was quantified across patients (right). Scale bars = 2 µm (upper) and 200 nm (lower). D) Picrosirius red staining under polarized light reveals collagen fiber architecture in Nby and Exp dermis. Quantification of average fiber length and porosity percentage is shown (right). Scale bar = 25 µm. E) Clinical information and molecular phenotypes of human skin expansion samples, including patient age, site, duration, and summarized trends in stem cell activity, COL17A1 expression, and ECM turnover. F) Schematic illustration of the structural and molecular changes during sufficient (left) vs exhausted (right) phases of mechanical stretch‐induced skin regeneration. RE is characterized by impaired stem cell activity, disrupted adhesion, increased ECM degradation, and mechanical instability, involving MMP2‐mediated COL17A1 proteolysis.

In clinical practice, the selection of anatomical donor sites and duration of skin expansion therapy vary significantly depending on preoperative diagnoses and surgical requirements, which complicates the systematic assessment of regenerative capacity over prolonged expansion.^[^ ^4^ ^]^ In this study, we analyzed paired skin samples—expanded (Exp) and nearby non‐expanded (Nby)—from five patients undergoing skin expansion procedures with differing demographics (age, sex) and expansion durations (**Figure** **6**E). Despite these variability, clonal analysis showed a general trend toward reduced clonogenicity and adhesion of IFESCs ex vivo in expanded skin compared to matched controls (Figure S7A, Supporting Information).

COL17A1 protein expression exhibited inter‐individual variability (Figure 6A); however, samples with lower COL17A1 levels were frequently associated with reduced proliferation and stemness markers (Figure 6B), suggesting a possible link between COL17A1 expression and regenerative competence. Ultrastructural assessment revealed variable degrees of HD shortening across cases (Figure 6C). In situ hybridization detected *MMP2* transcript accumulation in the dermis of expanded regions (Figure S7B, Supporting Information), consistent with the mechanosensitive expression patterns observed in the mouse model. Furthermore, samples with reduced COL17A1 levels also exhibited shorter fibrillar lengths and increased collagen porosity, indicative of increased collagen degradation and fragmentation (Figure 6D).

While the number of human samples remains limited, these findings suggest that COL17A1 may serve as a potential marker of both epidermal regenerative status and dermal collagen remodeling under mechanical stress. Its reduction may reflect early signs of mechanical overloading and impending RE; however, further studies with larger patient cohorts and functional validation are needed to determine its clinical relevance and utility in guiding expansion protocols.

Collectively, our findings identify excessive mechanical stress and MMP2‐mediated proteolysis as key pathological drivers that disrupt both dermal ECM integrity and basal epithelial adhesion, thereby promoting RE. Importantly, pharmacological inhibition of proteases using agents like Marimastat could potentially preserve COL17A1 and dermal collagen, offering a therapeutic avenue to sustain tissue regeneration during prolonged mechanical expansion (Figure 6F).

## 3 Discussion

Mammalian tissues have limited regenerative capacity, which impedes full restoration of structure and function following injury or chronic stress.^[^ ^93^ ^]^ Understanding the mechanisms of RE is crucial for developing strategies to preserve tissue repair potential.^[^ ^15^ ^]^ In this study, we used a mouse scalp expansion model with cell‐type‐specific resolution and time‐series analyses to explore the mechanistic basis of RE induced by LTE. Our findings uncover a novel cross‐compartmental pathway of mechanical signal propagation and disruption, which ultimately impairs epithelial stem cell function and tissue regeneration.

We found that LTE triggers non‐homogeneous responses across skin compartments, largely due to intrinsic differences in cell lineage and mechanical behavior.^[^ ^18^, ^32^ ^]^ As LTE progresses, progressive accumulation of MMP2 in dermal fibroblasts led to excessive degradation of ECM collagen fibers, reducing substrate stiffness and force dissipation capacity within the stem cell niche. These changes further initiate COL17A1 proteolysis and compromise BM integrity. These structural and molecular alterations reshape the local microenvironment of IFESCs, subsequently impairing epithelial cell division, extrusion, and altering their morphology. These findings underscore the critical need for coordinated actions among skin compartments to maintain tissue homeostasis, as any disruption in this balance may negatively impact tissue regeneration and remodeling.

Importantly, our ultrastructural analyses identified the degradation of the BM and HDs as central events linking dermal ECM remodeling to epidermal RE. In the skin, type I HDs are multiprotein complexes that anchor basal keratinocytes to the BM, comprising integrin α6β4, the transmembrane collagen COL17A1 (also known as BP180), the tetraspanin CD151, and cytoskeletal linker proteins plectin and BPAG1e (BP230).^[^ ^80^, ^94^, ^95^ ^]^ Among these components, COL17A1 serves as a structural bridge by spanning the plasma membrane—linking intracellular keratin intermediate filaments to extracellular BM elements, including laminin 332 and type IV collagen.^[^ ^21^ ^]^ Notably, COL17A1 has been shown to be mechanosensitive in ex vivo models, with its expression modulated by mechanical stretch,^[^ ^96^ ^]^ highlighting its potential vulnerability under long‐term expansion.

Structurally, COL17A1 is a type II transmembrane collagen with a long extracellular ectodomain that includes 16 non‐collagenous (NC) domains, notably the juxtamembranous NC16A region, which is particularly susceptible to proteolytic cleavage.^[^ ^85^, ^97^, ^98^ ^]^ This inherent vulnerability makes COL17A1 one of the most proteolytically unstable components of the HD complex.^[^ ^80^, ^83^ ^]^

In our LTE model, MMP2 progressively accumulated at the dermal–epidermal junction and contributed to targeted degradation of COL17A1. As a gelatinase, MMP2 cleaves both type I and IV collagen, thereby disrupting the structural integrity of both the dermal ECM and the BM.^[^ ^99^, ^100^ ^]^ This dual substrate specificity may explain the coordinated structural failure across skin compartments. Previous studies in Drosophila^[^ ^87^ ^]^ and human skin disorders, such as vitiligo,^[^ ^88^ ^]^ have similarly implicated MMP2 in ECM destabilization and loss of epithelial adhesion. Furthermore, studies using ex vivo human skin equivalent models also support that elevated MMP2 levels cause collagen fragmentation, ECM degradation, and dislocation of BM components that bind to epidermal integrins, ultimately impairing epithelial stem cell preservation and stemness.^[^ ^101^ ^]^

As mechanical expansion progressed, increased intercellular spacing led to a gradual reduction in HD density and width. Once HD density and adhesive strength fell below a critical threshold, basal keratinocytes could no longer maintain attachment to the BM, resulting in cellular detachment, flattening, and impaired regenerative behavior. TEM analysis confirmed this cascade, revealing progressive HD loss, BM micro‐delamination, and basal epithelium disorganization. These structural defects directly impaired epidermal mechanotransduction and disrupted IFESC homeostasis.

Collectively, our data support a model in which MMP2‐mediated ECM degradation initiates a biomechanical failure cascade—characterized by tissue softening, BM disruption, COL17A1 proteolysis, and ultimately RE. This degradation process is progressive and most pronounced at later stages of expansion (e.g., DPE36), supporting a cumulative damage model. These findings reinforce the concept of a biomechanical “yield strength” in skin regeneration—a critical threshold determined by the magnitude and duration of mechanical force, beyond which the tissue's adaptive capacity is overwhelmed.^[^ ^32^, ^102^, ^103^ ^]^ While short‐term expansion preserves dermal‐epidermal integrity and regenerative capacity, prolonged mechanical stress fractures collagen and elastic fibers and disrupts cell–matrix adhesions. Once this critical threshold is surpassed, regenerative exhaustion becomes irreversible.

Clinically, our findings offer practical guidance for optimizing tissue expansion protocols to prevent RE. Both prolonged expansion duration and overly frequent inflations contribute to RE via distinct mechanisms—frequent inflations risk acute tissue damage, while extended duration leads to gradual regenerative decline. Notably, more frequent inflations transiently delayed RE onset, supporting that RE results from cumulative stress over time rather than immediate strain. These results underscore the effective inflation rate—a balance of volume and timing—as a key factor in preserving regenerative potential. Furthermore, our deflation experiment demonstrates that LTE‐induced impairments are largely irreversible, even after mechanical tension is withdrawn. These results reinforce the concept that RE reflects a true and sustained loss of regenerative capacity, rather than a transient adaptive response. Together, these findings highlight the need for close monitoring of regenerative status during prolonged expansion and the importance of timely intervention before irreversible damage occurs. Future studies are warranted to determine whether skin subjected to LTE exhibits increased susceptibility to pathological remodeling, compromised graft quality, or delayed wound healing following transplantation.

Therapeutically, our study proposes a promising intervention strategy against LTE‐induced RE. We demonstrate that intradermal administration of the MMP inhibitor Marimastat—originally developed for anticancer applications^[^ ^75^, ^90^ ^]^—effectively preserves tissue integrity under mechanical stress. Compared to systemic delivery, intradermal injection offers a clinically feasible route with reduced risk of off‐target effects commonly associated with Marimastat's broad‐spectrum activity. Similar intradermal approaches using bone marrow‐derived mesenchymal stem cells (BM‐MSCs),^[^ ^3^ ^]^ mononuclear cells (MNCs),^[^ ^10^ ^]^ or stromal vascular fraction cells (SVFs)^[^ ^104^ ^]^—have shown efficacy in mitigating RE and enhancing dermal volume in clinical settings. In our model, Marimastat treatment stabilized COL17A1 protein levels, preserved HD and BM architecture, restored collagen ultrastructure, and improved IFESC clonogenicity. These findings support the repurposing of Marimastat as a targeted anti‐degradative agent for managing mechanically induced skin degeneration, including chronic wounds,^[^ ^105^, ^106^ ^]^ and aging‐associated tissue remodeling.^[^ ^107^ ^]^ Furthermore, given emerging evidence that stromal stem cell therapies may exert beneficial effects through MMP modulation,^[^ ^108^, ^109^ ^]^ future studies should explore the therapeutic synergy between MMP inhibition and cell‐based approaches.

### 3.1 Limitations of the Study

This study provides insights into a 3‐stage process of the epidermal mechanoregenerative response, encompassing initiation, maximization, and ultimately exhaustion, which align with the early, middle, and late phases of stretch‐induced regeneration. While this framework offers a foundational perspective, our transcriptomic analysis may not fully capture the dynamic gene expressions occurring during the peak phase of mechanoregenerative activity.

In clinical settings, skin expansion therapy presents a complex challenge that requires precise monitoring to achieve optimal outcomes.^[^ ^110^, ^111^ ^]^ While the expansion phenotypes in mice are well characterized, human responses necessitate careful, quantitative analysis. Our findings highlight the importance of larger patient cohorts and extended longitudinal follow‐up to effectively track regenerative capacity over time. These approaches would allow for a dynamic assessment of patient responses to treatment, enhancing clinical decision‐making and treatment personalization.

Furthermore, it is essential to delve into the safety of Marimastat treatment to avoid surgical failure related to RE in the clinical treatment.^[^ ^90^ ^]^ Future studies should further evaluate Marimastat's safety profile, specifically examining its long‐term impact on mechanoregenerative function and overall treatment efficacy.

## 4 Conclusion

Our study concludes that long‐term tissue expansion transitions interfollicular epidermal stem cells from regenerative sufficiency to exhaustion, accompanied by a shift in dermal extracellular matrix (ECM) turnover from collagen deposition to degradation. Accumulated dermal matrix metalloproteinase 2 (MMP2) plays a central role in this process by mediating trans‐layer proteolysis of both dermal collagen and the epidermal hemidesmosomal protein COL17A1, thereby disrupting niche integrity and impairing stem cell function. This cascade culminates in irreversible regenerative exhaustion (RE). Treatment with the protease inhibitor Marimastat effectively mitigated collagen degradation, preserved COL17A1 levels, and delayed RE onset. Additionally, COL17A1 was validated as a clinical marker of ECM integrity and regenerative potential in human expanded skin, indicating possible relevance of this pathway in clinical contexts. These findings provide a mechanistic framework for understanding stretch‐induced regenerative decline and may inform the refinement of tissue expansion protocols.

## 5 Experimental Section

### Human Skin Samples

Human expanded tissue samples were obtained from discarded specimens following full facial reconstruction with autologous tissue transplantation at Shanghai 9th People's Hospital (Shanghai, China). Individuals who underwent tissue expansion for more than five months were included in the study (*n* = 5) and sex was not considered as a biological variable. Detailed patient information was provided in Table S4 (Supporting Information). The study was conducted with written informed consent from all participants and was approved by the Ethical Review Board of Shanghai 9th People's Hospital, Shanghai Jiao Tong University School of Medicine (Approval No. 2020‐2018‐129‐T107‐1). All procedures adhered to the ethical standards outlined in the Helsinki Declaration (last revised in 1983).

### Animals

All animal experiments were approved by the Institutional Animal Care and Use Committee (IACUC) of the Shanghai Institute of Nutrition and Health, Chinese Academy of Sciences (Approval No. SINH‐2020‐ZL‐1). All animals were housed under specific‐pathogen‐free (SPF) conditions. Only male mice were used in this study to reduce variability due to sex‐related differences. For the generation of the *R26‐CAG‐LSL‐Col17a1* mouse line, the *CAG‐loxp‐stop‐loxp‐Col17a1(CDS)* sequence was inserted into the *Rosa26* locus of C57BL/6J mice by Cyagen Biosciences Inc. The *Krt14‐CreER;Rosa26‐mTmG* mice, essential for lineage tracing studies, were generously provided by Dr. T.Chen from the National Institute of Biological Sciences. Mouse genotyping was performed with genomic DNA isolated from tail biopsies, and the primers used are shown in Table S5 (Supporting Information). For *C* *ol17a1* overexpression studies, tamoxifen (Sigma‐Aldrich) was dissolved in corn oil (Mercklin) at a final concentration of 10 mg mL^−1^ and sterilized using a 0.22 µm syringe filter (Millipore Sigma) for intraperitoneal administration. Tamoxifen was administered at a dosage of 50 mg kg^−1^ over five days to induce COL17A1 expression. In low‐dose tamoxifen clone tracing experiments, mice administered tamoxifen at 2 mg kg^−1^ via intraperitoneal injection, enabling visualization of single basal stem cells and tracking labeled clones over 8‐day course. Throughout the experimental procedure, mouse body weight was measured at key intervals: before expander insertion, immediately after the insertion, and after each saline infusion. The final body weight was adjusted by subtracting the weight of the expander device and the infused saline. The tamoxifen injection dose was calculated based on this net volumetric weight.

### Standard Paradigm for Mouse Expansion Model

Male C57BL/6J mice (7–8 weeks old, 22–25 g) were obtained from Shanghai SLAC Laboratory Animal Co., Ltd. All animal procedures were approved by the institutional ethical committee. Animals were not randomized, and investigators were not blinded to group allocation. All animals used were of similar age (7‐8 weeks old) and weight (22–25 g). Prior to the surgical procedure, mice were anesthetized using isoflurane to ensure humane treatment. The dorsal skin was thoroughly disinfected with 75% alcohol to minimize the risk of postoperative infection. A 1 cm transverse incision was made in the cervical dorsal skin, followed by the creation of a subcutaneous pocket using forceps, extending both cranially and caudally. A 1 mL silicone expander (Wanhe Plastic Materials Co., Ltd, Catalog No. 00 02 621), sterilized with cyclohexane, was implanted beneath the scalp. In the experimental group, tissue expansion was initiated by injecting 0.6 mL of PBS into the expander. The incision was then sutured.

To establish the LTE model, PBS was injected into the expander at specified intervals to gradually increase mechanical load. Two injection regimens were used for comparison: a 2‐day interval group, in which 0.3 mL PBS was injected every 2 days, and a 4‐day interval group, in which 0.3 mL PBS was injected every 4 days. Both regimens reached a total volume of 3.0 mL and the day of expander implantation was designated as day 0. Skin samples were harvested on specified days post‐expansion (DPE) to assess tissue responses. The 4‐day interval protocol was adopted for subsequent experiments due to superior tissue tolerance and lower risk of skin necrosis.

To examine the reversibility of RE, a deflation experiment was conducted. After reaching 3.0 mL total volume by day 36, mice were randomly divided into two groups: a sustained tension group (Sus), in which 3.0 mL PBS was maintained; and a deflation group (Defl), in which 2.4 mL PBS was withdrawn through the valve of the implanted expander, without removing the device. This approach removed mechanical load while avoiding additional surgical injury. Skin samples were harvested on day 44 (8 days after deflation) from both groups for subsequent histological, molecular, and ultrastructural analyses

All analyses focused on the skin overlying the apex of the expansion dome, which experienced the most consistent and stable mechanical stress. Control group mice underwent identical surgical procedures, except without expander implantation or PBS injection, and served as baseline controls.

### Quantification of the Expansion Index

To objectively evaluate the efficiency of skin expansion, the Expansion Index was established, a metric based on the inverse relationship between HF density and skin surface area. Since HFs were fixed anatomical structures that did not regenerate, their absolute number remained constant during skin expansion, while their density decreased as the surface area increased. Thus, the expansion ratio was defined as the reciprocal of HF density (1/α), where α represented the HF density per unit area. HF density was quantified using a UV‐enhanced imaging system (CBS‐802, Taiwan CBS) under 50 × magnification. Under UV light, HFs fluoresced, allowing reliable counting in expanded skin (Exp) and untreated scalp (Ctrl). The expander was geometrically modeled as a hemisphere, with volume V = (2/3)πr^3^ and surface area A  =  2πr^2^, where r was the expander's radius. While saline injection volume was a directly measurable parameter, it correlated with volume increase. However, what affected skin expansion and HF density was the surface area. After raising the injected saline volume to the power of 2/3—effectively converting it from a volume‐based measurement to an area‐related estimate, this value was then normalized to the initial volume at day 4 (DPE4) to allow temporal comparison to derive theoretical curve. In contrast, the empirical expansion index was calculated based on measured HF density at each time point. Specifically, the reciprocal of HF density (1/α) was used—proportional to the expanded area—and normalized it to the baseline value at DPE4. This provided a dynamic index reflecting actual tissue expansion over time. Control animals underwent the same HF quantification without tension, generating a control expansion curve (Ctrl). This comparative analysis between the experimental and theoretical groups allowed for a thorough evaluation of the skin expansion process.

### Quantitative Analysis of Mouse Collagen Ultrastructure

For quantitative analysis of Picrosirius Red‐stained sections, expanded and normal skin samples were examined from ≥ 5 biological replicates per condition. To establish standardized benchmarks for extreme ECM remodeling states, two well‐characterized biological comparators were incorporated: 1) stretch‐induced wound fibrosis samples representing pathological collagen deposition,^[^ ^71^ ^]^ and 2) 24‐month‐old murine dermis exemplifying age‐related collagen degradation.^[^ ^27^ ^]^

Briefly, deparaffinized sections were incubated in picrosirius red staining solution (0.1% Sirius red [w/v] in saturated picric acid) for 1 h and then washed for 5 min with acidified water (0.5% acetic acid [w/v]). The light intensity was adjusted to obtain a linear response for quantification. All sections were imaged in the longitudinal direction. The same microscope settings, including the illumination, exposure, saturation, and degree of polarization, were maintained throughout the analysis. Next, color deconvolution of Picrosirius Red images were performed in ImageJ using the previously published algorithm.^[^ ^70^, ^71^ ^]^ wherein each pure stain is characterized by absorbances within three RGB channels (Color 1 = [1 0 0], Color 2 = [0 1 0], Color 3 = [1 1 1]). Ortho‐normal transformation of the histology images produced individual images corresponding to each color's contribution to the image. Applied to birefringent Picrosirius Red images (green to red color under polarized light depending on the packing of fiber bundles), this technique produced deconvoluted red and green images corresponding to mature collagen and immature collage fibers, which were then analyzed independently. Analysis was thus performed purely using ECM fibers, with no cellular elements included. Noise reduction of deconvoluted fibers was achieved using an adaptive Wiener filter in Matlab 2019a (wiener2 function), which tailored itself to the local image variance within a pre‐specified neighborhood (3‐by‐3 pixels in the application). The filter preferentially smoothed regions with low variance, thereby preserving the sharp edges of fibers. Smooth images were then binarized using the im2bw command and processed through erosion and dilation filters with both linear and diamond‐shaped structuring elements to select fiber‐shaped objects. Finally, the fiber network was “skeletonized” using the bimorph command and various parameters of the digitized map (Brightness, Number of Fibers, fiber length, width, persistence, Angle Randomness, Number of Branchpoints, Euler Number, Extent, Perimeter, Solidity, Eccentricity, and Equivalent Diameter) were measured using the regionprops command. Dimensionality reduction of quantified fiber network properties by Uniform Manifold Approximation and Projection (UMAP) was achieved using the Umap package in R.

### ECM Turnover Scoring Model

ECM turnover scores were assigned by setting the non‐stretched control group as 0 and the fibrosis group, which represented the gold standard in collagen morphology, as 1. This scoring function was established to evaluate the collagen turnover balance from deposition to degradation. Feature selection was performed using LASSO regularization^[^ ^112^ ^]^ with the cv.glmnet function from the glmnet package in R, applied to a set of 26 collagen ultrastructure parameters.^[^ ^71^ ^]^ A multiple linear regression model was subsequently used to derive the ECM turnover scoring model.

### Collagen Morphometric Analysis in Human Samples

Given interspecies morphological variability and the limited sample size of the human dataset (case‐control only), a simplified and clinically interpretable workflow was employed for collagen morphometric analysis. Images were analyzed using ImageJ software. RGB channels were separated, inverted, and normalized using cumulative histogram equalization. Collagen fiber segmentation was performed using the Segment function within the DiameterJ plugin (v1.018),^[^ ^113^ ^]^ with default parameter settings. Quantification of structural features—including fiber length and porosity percentage—was conducted based on the segmented images. All measurements were calibrated to micrometer scale using the corresponding image scale bars.

### Full‐Thickness Human Skin Equivalents Model

A Full‐thickness human skin equivalents model was constructed as previously described.^[^ ^114^ ^]^ Briefly, the *MMP2*‐OE and ctrl HFFs were mixed in a collagen gel solution at a density of 4 × 10^6^ cells mL^−1^, which consisted of 80% 3 mg mL^−1^ rat tail collagen (Thermo Fisher Scientific), 10% 10 × PBS, 10% FBS and 0.025 mm NaOH. A 400 µL aliquot of this fibroblast/gel mixture was placed onto each 0.4 µm pore size insert in a 12‐well plate (Biofil) and incubated at 37 °C for 1 h to facilitate polymerization. The inserts were then coated with 25 µL of 50 µg mL^−1^ fibronectin for 30 min before the addition of 4 × 10^5^ HFKs suspended in 0.5 mL of keratinocyte medium (8 × 10^5^ cells mL^−1^) onto each fibroblast/collagen gel bed. An additional 2 mL of keratinocyte medium was added to the outer well to equalize the liquid level. The culture was incubated at 37 °C for 2–3 days. After the initial incubation, the medium was removed from the inserts to lift them to the air‐liquid interface, and Epilife Differentiation Medium was added to the outer well, reaching the insert membrane. This medium was prepared by supplementing Epilife Medium with 1.15 mm CaCl_2_, 50 µg mL^−1^ L‐ascorbic acid, 0.1% bovine serum albumin, and 10 µg mL^−1^ transferrin. The skin cultures were further incubated at 37 °C for an additional 10 days before being harvested.

### In Vitro Protease Inhibitor Drug Screening Assay

For in vitro drug screening assay, HFKs at passage 5 and MKs at passage 2 were seeded into cell culture plates at equivalent densities. A collection of 59 small molecule protease inhibitors (refer to Table S3, Supporting Information), sourced from TargetMol, Inc., was added to the cultures at a concentration of 6 µm for 24 h. To inhibit de novo protein synthesis, 10 µm Cycloheximide (CHX, Sigma) for human keratinocytes and 3 µm CHX for mouse keratinocytes were introduced into the culture medium 17 h prior to cell collection.^[^ ^83^ ^]^ After treatment, keratinocytes were subjected to western blotting analysis to evaluate the impact of the protease inhibitors on COL17A1 protein expression levels.

### Mouse Drug Intradermal Deliver Assay

The in vivo drug delivery assay was conducted on mice from DPE20 to DPE36. Mice were anesthetized, and their skin surfaces were disinfected with 75% alcohol. Intradermal (ID) injections were administered at a 15‐degree angle to the skin plane using microneedles (Hamilton, 21–2065) at multiple sites every two days and should have been avoided puncturing the underlying expander. The experimental drug, Marimastat (Sigma), was initially dissolved in DMSO at a concentration of 10 mg mL^−1^ and then diluted with PBS to a final concentration of 100 µg mL^−1^. Control injections consisted of equivalent volumes of DMSO and PBS solutions.

### Cell Isolation

The isolation of skin cells was conducted based on a modified protocol from previous studies.^[^ ^53^ ^]^ Briefly, both expanded and control mouse skin samples were trimmed of hair using scissors. The skin was isolated and floated on ice‐cold HBSS (Thermofisher, 14 175 079) supplemented with 0.04% BSA (Yeasen). Subsequently, the subcutaneous fascia and adipose tissue were meticulously excised using a scalpel. The skin was then minced into small pieces, ≈1 mm in width, and subjected to enzymatic digestion in 0.02% collagenase type I (ThermoFisher, 17 100 017) solution at 37 °C for 1 h. This treatment yielded a partially digested single‐cell suspension, predominantly comprising stromal cells, which was obtained through serial pipetting. This suspension was then strained through a 40 µm filter, and the filtrate was collected and stored on ice. The residual undigested tissue on the cell strainer was further treated with 0.05% trypsin‐EDTA (Gibco, 25300054) for 15 min at 37 °C. The resulting dissociated cells, primarily consisting of epidermal cells, were pipetted, filtered through the 40 µm strainer and collected via centrifugation at 500 g for 5 min at 4 °C. Following erythrocyte lysis, the viability of the cell suspension was assessed using Countess II Automated Cell Counter. The resulting single‐cell suspensions were then prepared for 10x Genomic sequencing. Each experimental time point (DPE0, DPE8, DPE36) was represented by samples from three individual mice, ensuring the reproducibility of the findings.

### Preliminary Processing of scRNA‐seq Raw Data from 10x Genomics

Transcripts were mapped to the mm10 reference genome using Cell Ranger (version 7.0.1). Seurat (version 4.1.0) was used for downstream quality control, integration, cell type identification, and differential gene expression analysis on the gene‐cell expression matrices obtained. DoubletFinder (version 2.0.3) was applied to detect and remove possible doublets from technical artifacts. Cells with fewer than 800 or more than 6000 detected genes, as well as those with a mitochondrial gene content greater than 10%, were excluded from further analysis.

Dimensionality reduction and clustering were conducted using 2600 highly variable genes and the first 40 principal components (PCs). Detailed instructions for Seurat analyses in this study were available in the Seurat tutorial. To determine the gene expression specificity within each cluster, the “FindAllMarkers” function was used with thresholds set to avg_log2FC > 0.5 and p_val_adj < 0.05, identifying highly expressed genes (marker genes) unique to each cluster and aiding in cell type assignment.

### Identification of L/E DEGs Across Tissues and Cell Compartments

The “FindMarkers” function in Seurat was used to identify differentially expressed genes (DEGs) in the late expansion stage (DPE36) vs the early expanded stage (DPE8) (L/E) of each cell cluster, which were based on normalized data and the Wilcoxon test. The screening criteria for DEGs were selected by BH‐adjusted p value < 0.05 and |log2FC| > 0.25.

### Gene Set Enrichment Analysis and Gene Ontology Analysis

For refined gene set enrichment analysis (GSEA) in single‐cell transcriptomics, the irGSEA package^[^ ^40^ ^]^ was used to generate enrichment score matrices, enabling comparisons between samples at different time points. This analysis leveraged several gene set databases, including hallmark and KEGG gene sets from the Molecular Signatures Database (MSigDB, version 7.4.1), as well as custom gene sets derived from the following datasets: GSE133648^[^ ^41^ ^]^ (epidermal natural aging), GSE142471^[^ ^42^ ^]^ (epidermal wounding), GSE70523^[^ ^43^ ^]^ (stemness), GSE188432^[^ ^61^ ^]^ (dermal wounding), CRA004660^[^ ^62^ ^]^ (fibroblast aging). The Cordenonsi YAP conserved signature was also sourced from MSigDB, with all genes listed in Table S6 (Supporting Information). To facilitate cross‐species comparison, human gene sets were converted to mouse homologs using the HUGO Gene Nomenclature Committee (HGNC) database. Additionally, gene ontology (GO) analysis of differentially expressed genes (DEGs) was conducted using the clusterProfiler package^115^ to identify relevant pathways and representative terms from top‐ranked gene sets. The initial DEG evaluation utilized the Wilcoxon rank‐sum test to highlight significant changes in gene expression between conditions. For differential expression analysis, the Wilcoxon test was employed to determine the significance of enrichment results.

### Statistical Analysis

All statistical analysis Data was presented as mean ±SEM. Statistical analyses were performed using GraphPad Prism (v9) with unpaired two‐tailed Student's *t*‐test or two‐way ANOVA with mixed‐effects analysis followed by Tukey's post hoc test as appropriate. For gene set score analysis, two‐sided Wilcoxon rank‐sum tests were applied. A *p*‐value threshold of < 0.05 was considered statistically significant. All statistical methods, error bar representations, sample sizes (*n*), and exact *p*‐values were specified in the respective figure legends.

## Conflict of Interest

The authors declare no conflict of interest.

## Author Contributions

Y.‐D.S., Q.‐L.Q., L.‐W.X., B.‐W.G. contributed equally to this work. Q.‐F.L., C.‐Y.L., L.Z., S.‐J.W. conceptualized this project and supervised the overall experiments. Y.‐D.S. performed the majority of the experiments unless otherwise specified. Q.‐L.Q. and Y.‐D.S. conducted the bioinformatics analysis of the scRNA‐seq data. Q.‐L.Q. also carried out the machine learning analysis of picrosirius red staining images. L.‐W.X. calculated the expansion index, established the 3D skin equivalent model, and conducted part of the cellular experiments. B.‐W. G., Y.L., Q.‐Y.F., X.C., and N.C. assisted with human sample collection. T.L. provided the Python script for cellular area quantification. J.‐Y.Z. provided consultation on statistical methods. Y.‐D.S. took the lead in writing the manuscript and performed revisions. L.Z. reviewed the edited manuscript.

## Supporting information



Supporting Information

Supplemental Table 1

Supplemental Table 2

Supplemental Table 3

Supplemental Table 4

Supplemental Table 5

Supplemental Table 6

## Data Availability

The data that support the findings of this study are openly available in Gene Expression Omnibus (GEO) database at https://www.ncbi.nlm.nih.gov/geo, reference number GSE281659.
