# Supplementary material for: Mechanical Stretch‐Induced Interlayer Coordination between MMP2 and COL17A1 Exacerbates Regenerative Exhaustion in Skin
Source: Adv Sci (Weinh). 2025 Sep 17;12(41):e11474. doi: 10.1002/advs.202511474 (PMC12591213; doi:10.1002/advs.202511474)
Supplement: Supplementary file 1 — Supporting Information [file ADVS-12-e11474-s004.docx]

**Supplementary Materials for**

Mechanical Stretch-Induced Interlayer Coordination Between MMP2 and COL17A1 Exacerbates Regenerative Exhaustion in Skin

**Authors**

*Yidan Sun, Qili Qian, Luwen Xu, Bowen Gao, Ting Li, Yin Li, Jiayi Zheng, Qiaoyu Fu, Xi Cheng, Nuo Chen, Sijia Wang^*^, Liang Zhang^*^, Caiyue Liu^*^, Qingfeng Li^*^.*

**The PDF file includes:**

Figures. S1 to S6 and legends

Materials and Methods

References (1-6)

**Supplementary Figures and legends**

**
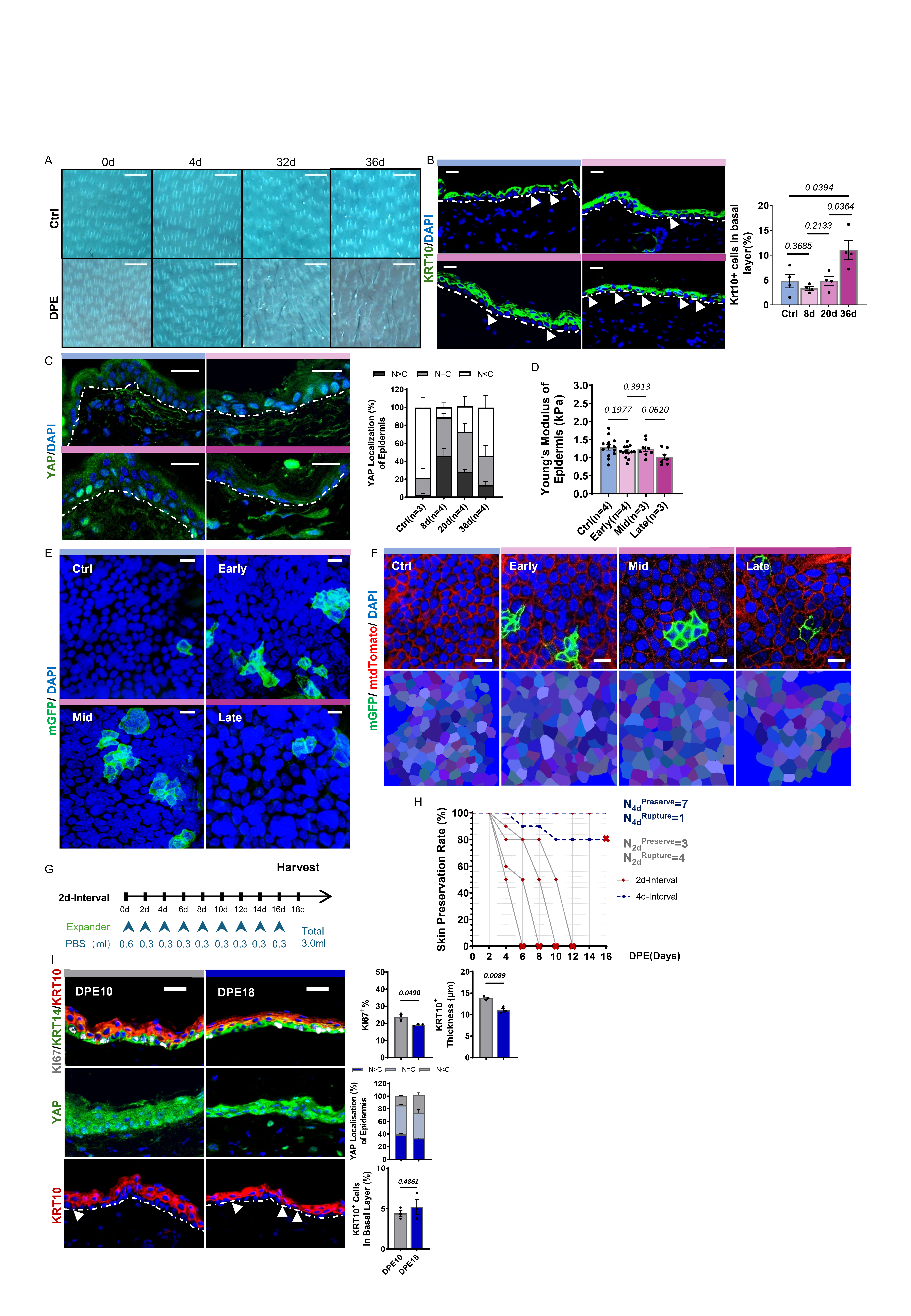
**

**Figure S1: LTE leads to exhaustion of epidermal regenerative capacity**

(A) Surface images of the expanded scalp and ctrl groups at different time points (d), with hair follicles appearing as lighter spots. Scale bar=500μm

(B) IF staining of KRT10 (green) in the epidermis, with arrows indicating KRT10^+^ basal cells. Scale bar = 50 μm.

(C) IF staining of YAP (green) and quantification of its subcellular localization in the epidermis at indicated expansion time points. Nuclear (N) > Cytosplasm (C), more YAP in nucleus than in cytoplasm, N = C, similar level of YAP1 in nucleus than in cytoplasm, N < C, less YAP in nucleus than in cytoplasm.

(D) Stiffness of the epidermis measured by AFM, represented as Young's Modulus (kPa). Data points indicate mean values derived from multiple stiffness curves per views (n ≥3 biological replicates per group).

(E) Cross-sectional views of clonal scans from cleared skin samples at different time points. Scale bar = 20 μm.

(F) Representative image showing automatic cell contour identification using the CellPose package. Scale bar = 20 μm.

(G) Schematic overview of the 2-day interval expansion protocol: (A) PBS injection regimen (arrows indicate 0.3 mL injections) until reaching final volume of 3.0 mL within 18 days.

(H) Temporal dynamics of skin preservation rate during tissue expansion with comparison of 2-day (grey) vs. 4-day (blue) injection intervals. Red crosses (✕) denote experimental termination due to skin rupture. Sample sizes: N_2d_=7 mice (4 ruptures, 3 preserved) showing higher rupture rate; N_4d_=8 mice (1 rupture, 7 preserved) indicating higher preservation rate.

(I) Representative IF staining of KI67 (white), KRT10 (red), KRT14 (green), YAP(green) and DAPI (blue) in 2-day interval expansion samples and their quantitative analysis.


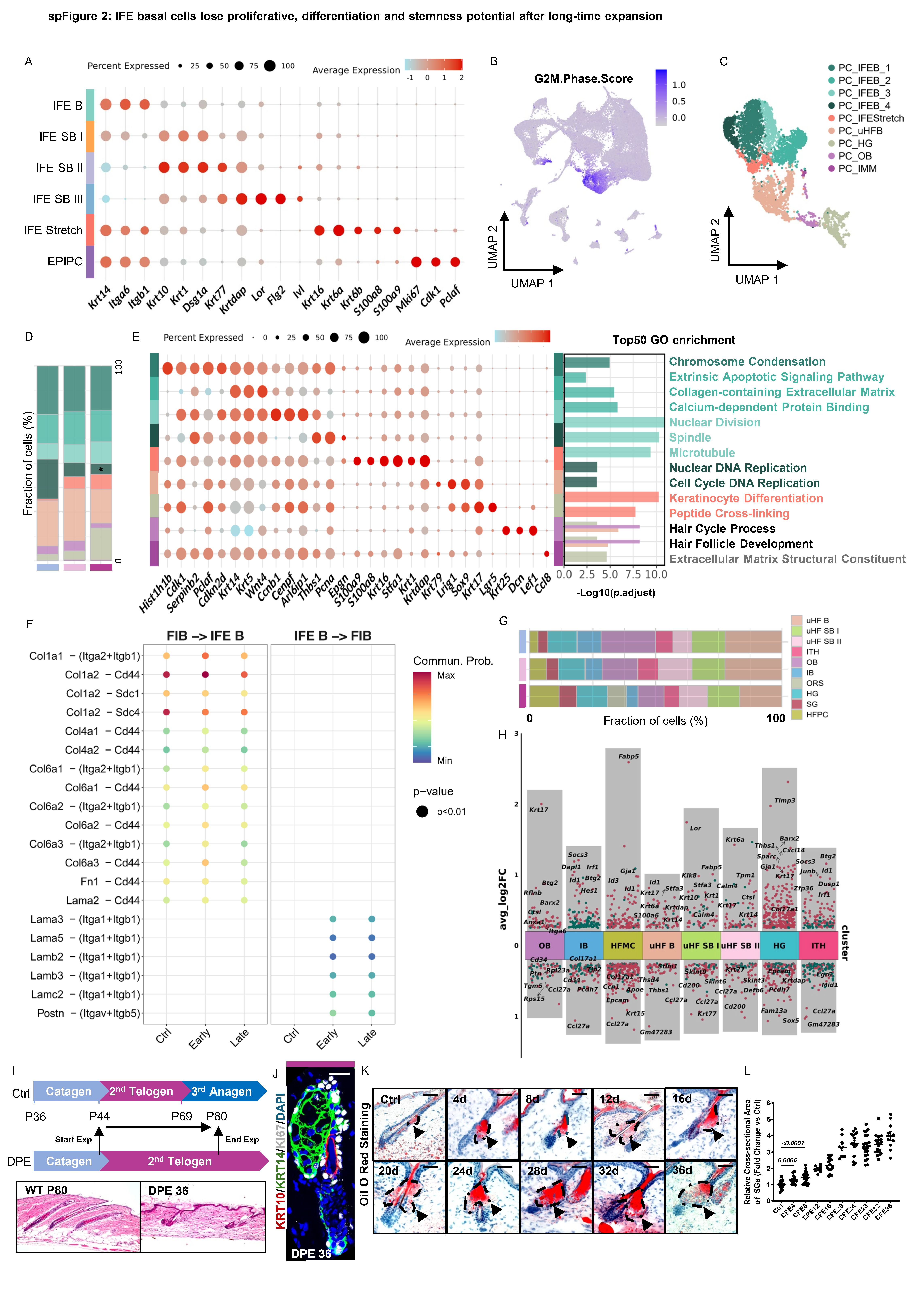


**Figure S2. IFE basal cells lose proliferative, differentiation and stemness potential after long-time expansion**

(A) Dot plots of marker genes for IFE clusters. The node size positively correlates with the percentage of a given type of cells positive for the given marker. The color key from green to red indicates low to high gene expression levels.

(B) UMAP plot showing cellular heterogeneity of proliferating cells (PCs).

(C) Feature plot showing the distribution of G2M enrichment score.

(D) Bar plots illustrate the composition of PC cell types in each group.

(E) Dot plots of marker genes in PC subclusters (left) and a bar plot showing cell-type-specific GO term enrichment (right). Node size correlates with the percentage of cells positive for a given marker. The color key (gray to red) indicates low to high gene expression.

(F) The dot plot reveals dynamic interlayer communication between FIB and IFE B during skin expansion using CellChat analysis, with communication probability and statistically significant interactions (*p*-value < 0.01) indicating robust cell-cell signaling.

(G) Bar plots showing HF lineage cell-type composition in each group.

(H) Differential gene expression analysis highlighting up- and down-regulated genes across HF lineage clusters between DPE36 and DPE8 samples.

(I) Schematic of hair cycle states in WT control and expanded mice at indicated time points (top), with H&E staining showing hair follicle status during expansion (bottom). Scale bar = 50μm.

(J) IF staining of KI67 (white), KRT10 (red), KRT14 (green), and DAPI (blue) in cross-sections of intact hair follicles at DPE36.

(K-L) Oil Red O staining of frozen sections at various time points shows sebaceous gland morphology, with arrows indicating cross-sectional views (K) and the corresponding quantification of cross-sectional area (L). Scale bar = 50 μm.


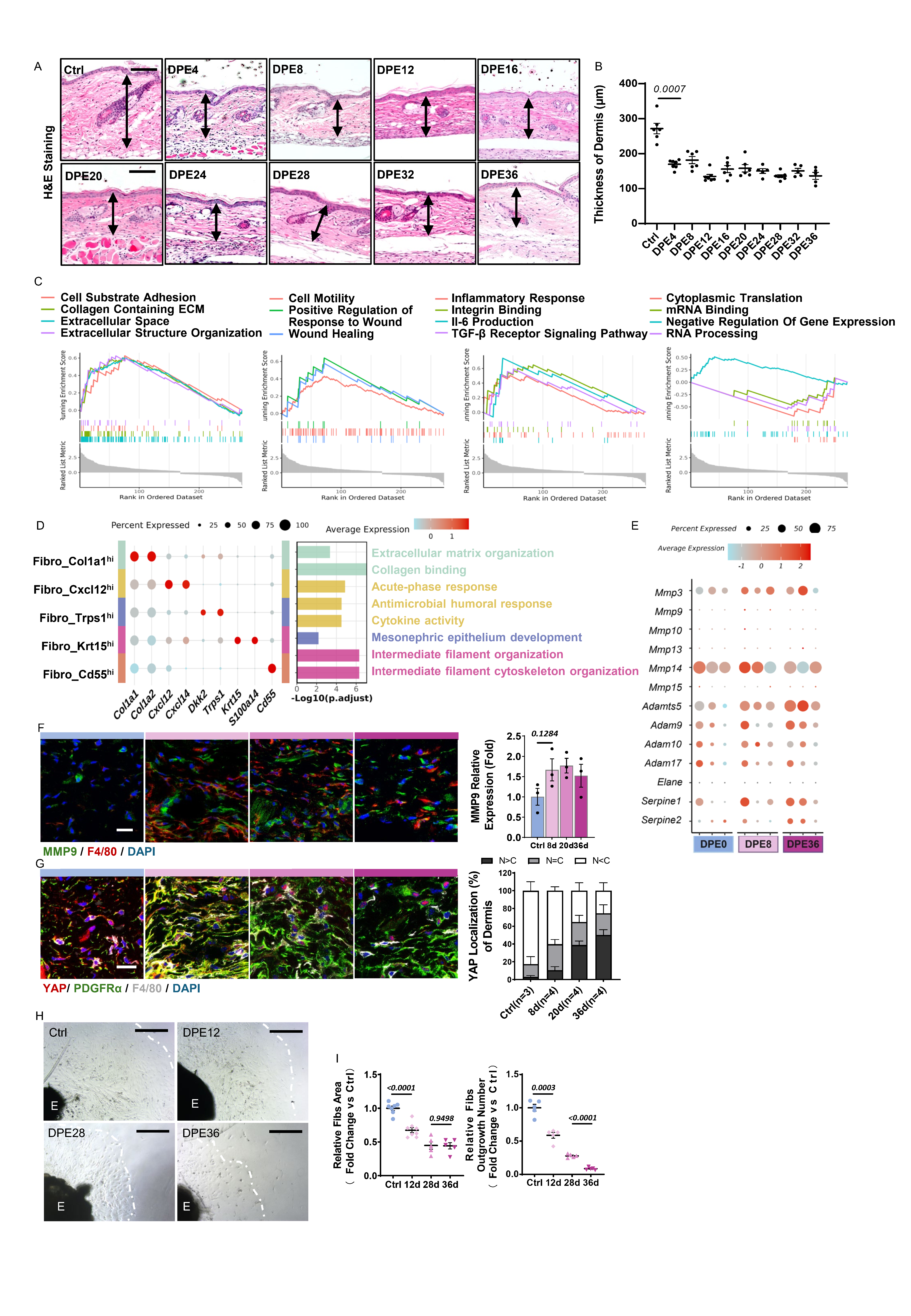


**Figure S3. LTE shifts collagen turnover towards degradation and promotes MMP2 accumulation in dermis.**

(A-B) H&E staining of dorsal skin across expansion time points (DPE: days post-expansion) and quantification of dermal thickness. Arrows indicate dermal layer. Scale bar = 100 μm. Each data point represents an individual mouse.

(C) Gene ontology (GO) enrichment plots of upregulated fibroblast genes in DPE36 versus DPE8 skin.

(D) Dot plot showing expression of marker genes across fibroblast subtypes (left), and bar graph summarizing subtype-specific GO term enrichment (right).

(E) Dot plot showing dynamic expression of proteinase-related genes (e.g., MMPs, ADAMs, Serpins) in fibroblasts across DPE0, DPE8, and DPE36.

(F) IF staining of dermal sections for MMP9 (green), F4/80 (red), and DAPI (blue) at indicated time points and corresponding quantification. *Each data point represents one mouse*. Scale bar = 20 μm.

(G) IF staining of YAP (red), PDGFRα (green), F4/80 (white), and DAPI (blue), with quantification of YAP subcellular localization categorized as nuclear-dominant (N > C), equal (N = C), or cytoplasmic-dominant (N < C). Each data point represents one mouse. Scale bar = 20 μm.

(H-I) Representative images and quantification of fibroblast outgrowth from control and expanded skin explants. Dashed lines indicate explant boundary. Quantified metrics include fibroblast migration area and migrated cell count. Quantification of migrated areas and number of migrated cells was performed using ImageJ. n≥ 3 biological replicates per group. E, explant. Scale bar = 500 μm.


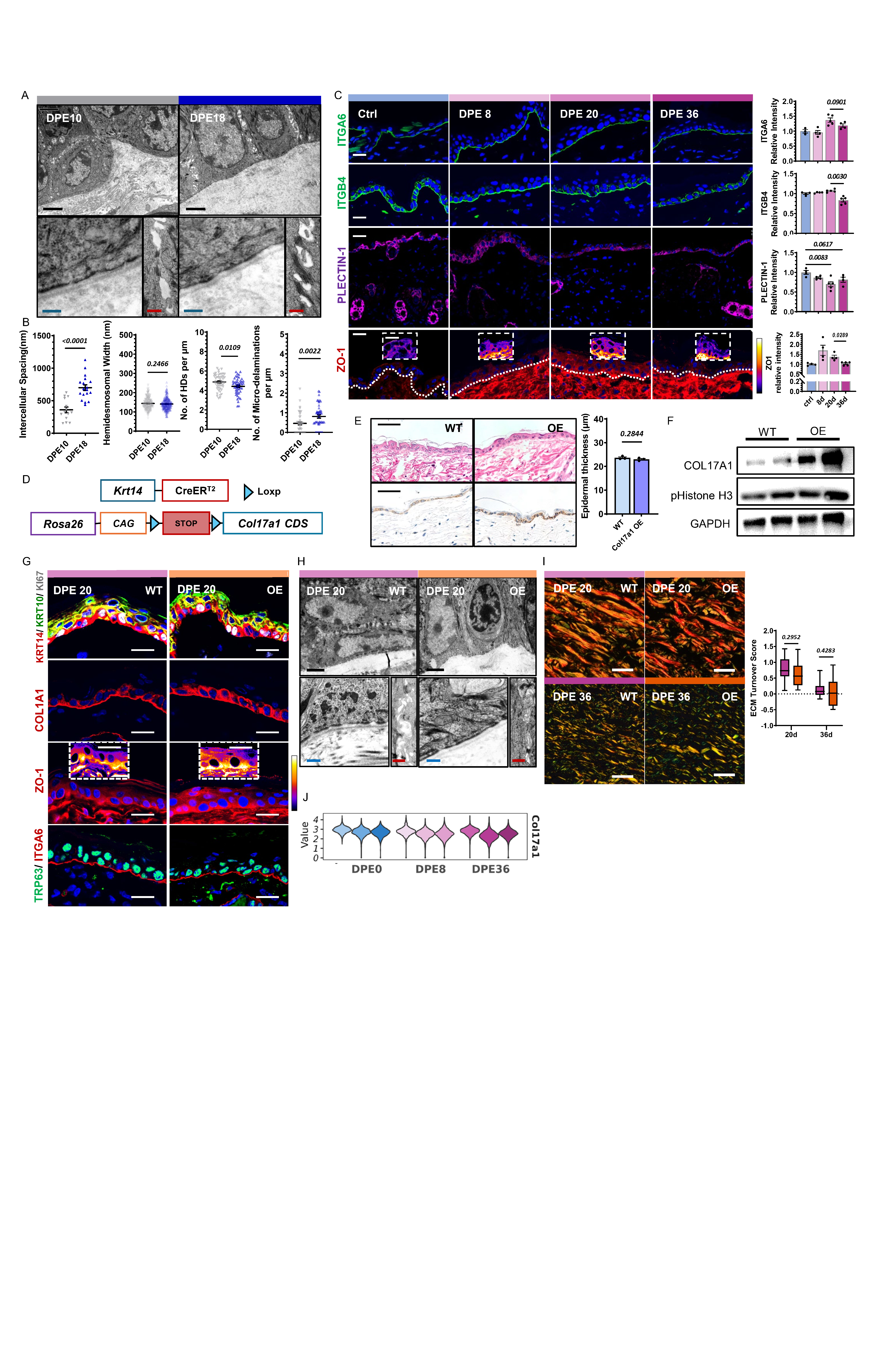


**Figure S4. Hemidesmosomes Breakdown of IFE Impairs Epidermal Regeneration and Epidermal *Col17a1* OE rescues LTE-RE**

(A) TEM images showing HD structure at DPE10 and DPE18. Scale bars: 2 µm (black), 200 nm (blue) and 500 nm (red).

(B) Quantification of intercellular spacing, HD length, number of HDs per μm, and number of micro-delamination at DPE10 vs. DPE18. Data are presented as mean ± SEM; *p*-values from unpaired *t*-tests.

(C) IF staining of ITGA6 (green), ITGB4 (green), PLECTIN-1 (purple), and ZO-1 (red) in epidermis at indicated timepoints. Quantification of fluorescence intensity is shown on the right. Scale bar = 20 µm.

(D) Schematic diagram of the genetic strategy used for *Col17a1* overexpression (OE) in Krt14⁺ basal epidermal cells using a *Rosa26-loxP-STOP-loxP* system.

(E) H&E staining of WT and *Col17a1*-OE skin under homeostatic conditions. Quantification of epidermal thickness is shown on the right. Scale bar= 50μm.

(F) WB analysis of COL17A1 and p-Histone H3 protein levels in epidermal lysates from WT and *Col17a1*-OE mice under homeostatic conditions.

(G) IF staining of KRT14 (green), KRT10 (red), Ki67 (white), COL17A1 (red), ZO-1 (red), TRP63 (green), and ITGA6 (red) at DPE20 in WT and *Col17a1*-OE mice. Scale bars = 20 μm.

(H) TEM images showing cellular adhesion ultrastructure in *Col17a1*-OE mice compared to WT at DPE20. Scale bars: 2 µm (black), 200 nm (blue) and 500 nm (red).

(I) Representative polarized light images of picrosirius red-stained dermal collagen in WT and *Col17a1*-OE mice at DPE20 and DPE36. ECM turnover scores are quantified on the right. Scale bars = 25 μm.

(J) Violin plots showing *Col17a1* mRNA expression dynamics in IFE B cells at DPE0, DPE8, and DPE36.


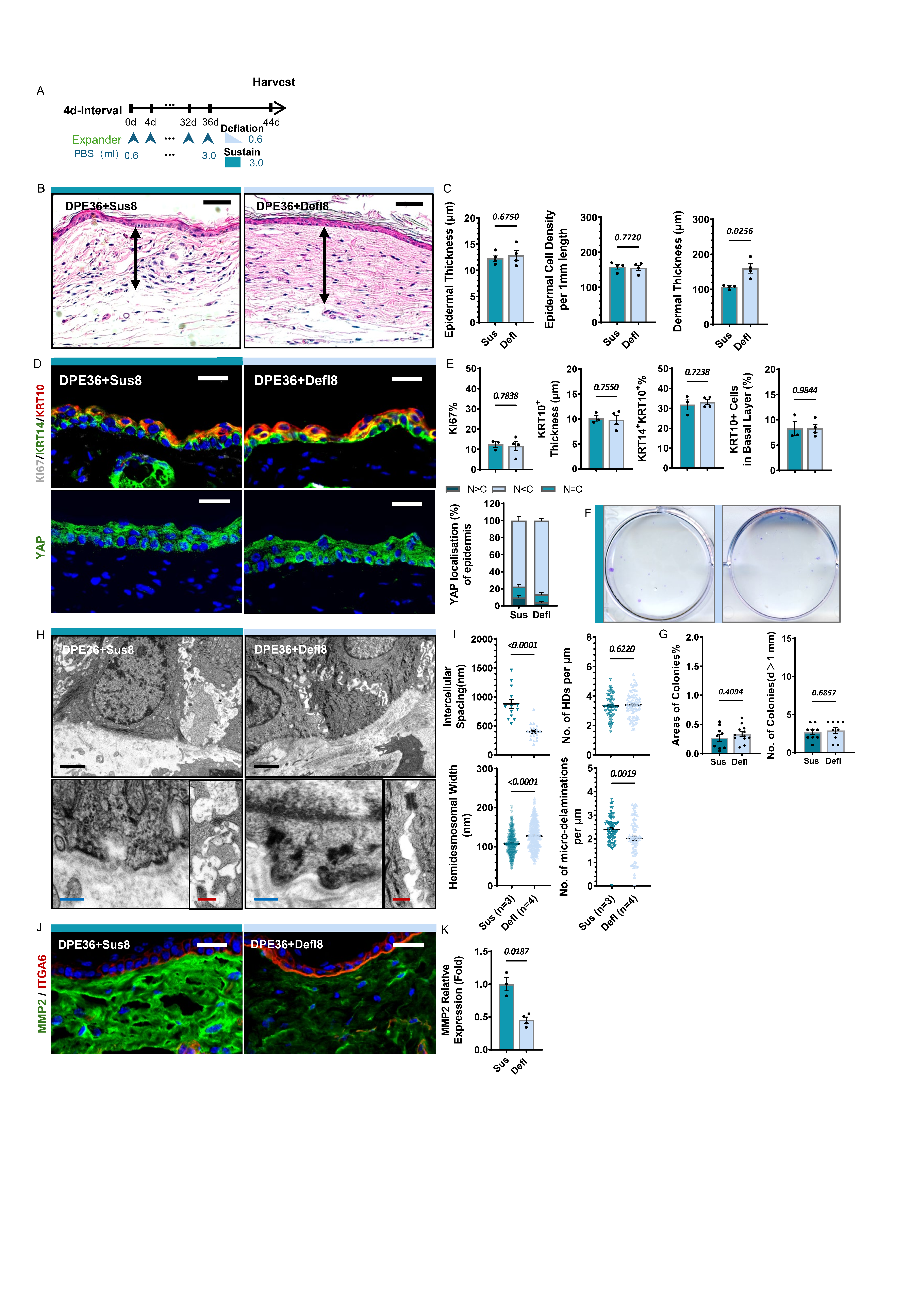


**Figure S5. Irreversible Loss of Epidermal Regeneration Despite stretch Removal**

**(A)** Schematic of the experimental protocol. Mice underwent skin expansion with PBS injections every 4 days, reaching DPE36. Two groups were then assigned: one underwent sustained expansion for 8 more days (Sus8), while the other underwent partial deflation on DPE36 followed by 8 days of rest (Defl8), and both were harvested at day 44.

**(B)** Representative H&E-stained images of skin sections from the Sus8 and Defl8 groups. Double-headed arrows indicate dermal thickness. Scale bars = 50 μm.

**(C)** Quantification of epidermal thickness, epidermal cell density per mm, and dermal thickness. Data are presented as mean ± SEM. *p*-values from unpaired *t*-tests.

**(D)** IF staining of KRT14 (green), KRT10 (red), and KI67 (white, upper panels), and YAP (green, lower panels) in Sus8 and Defl8 epidermis. Scale bars = 20 μm.

**(E)** Quantification of KI67⁺ ratio, basal layer KRT14⁺ thickness, KRT14⁺/KRT10⁺ double-positive cell percentage, and basal layer KRT10⁺ cell percentage. YAP nuclear localization (% of cells showing nuclear + cytoplasmic YAP) is also shown (lower left).

**(F)** Representative colony formation assay of keratinocytes isolated from Sus8 and Defl8 epidermis.

**(G)** Quantification of colony area and number of colonies >1 mm in diameter.

**(H)** TEM images showing ultrastructural features of epidermal adhesion. Images show HDs and micro-delamination structures at higher magnification. Scale bars: 2 µm (black), 200 nm (blue) and 500 nm (red).

**(I)** Quantification of intercellular spacing, hemidesmosome width, density of HDs and micro-delamination per μm basal membrane.

**(J)** IF staining of MMP2 (green) in Sus8 and Defl8 skin. Scale bar = 20 μm.

**(K)** Quantification of MMP2 fluorescence intensity in the dermis.


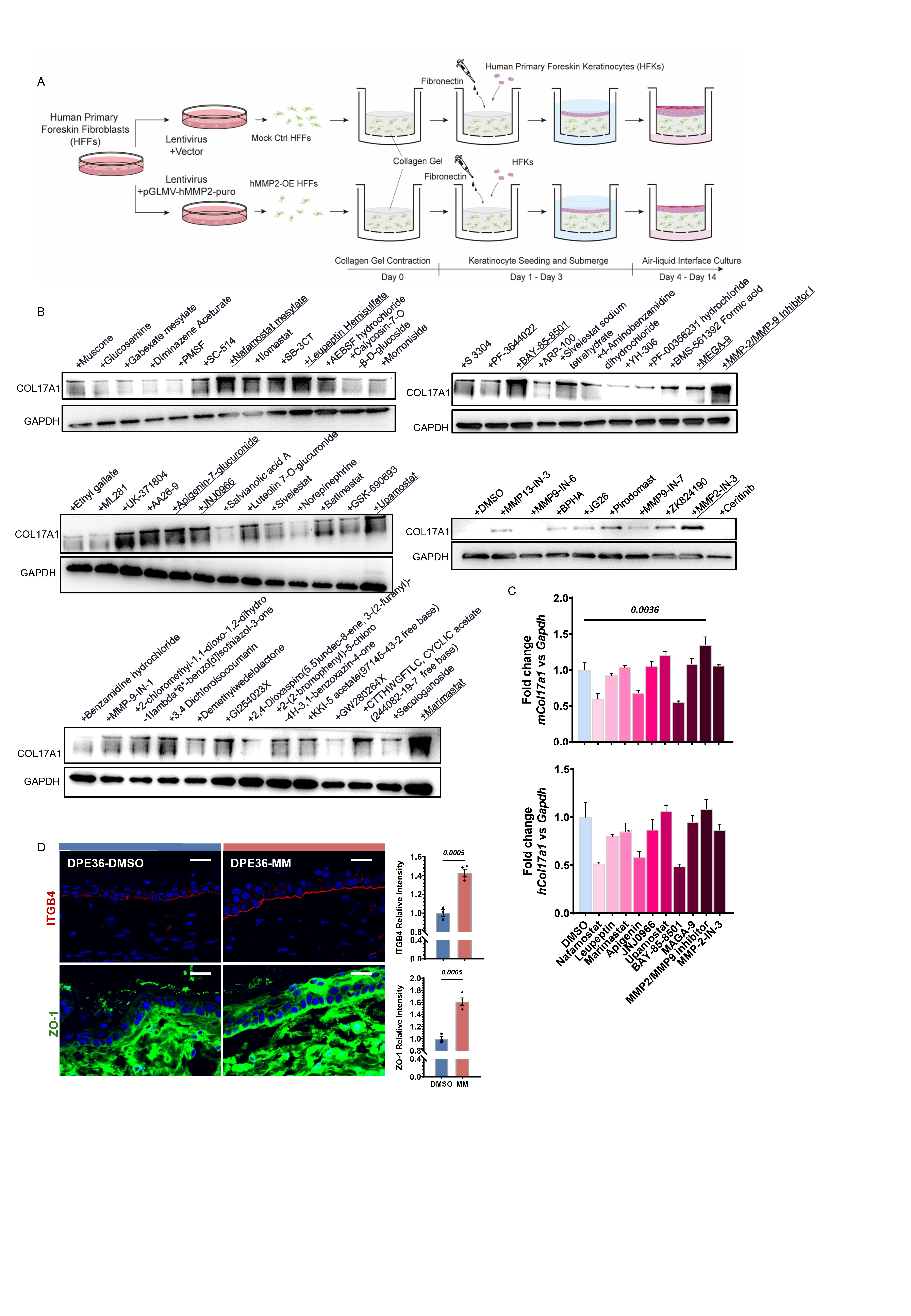


**Figure S6. MMP2 mediated dermal collagen degradation and COL17A1 proteolysis *in vitro*, and Marimastat restores dermal structure and rescue LTE-RE *in vivo***

1. Flowchart of the organotypic human skin equivalent culture process.
2. Western blots analysis of COL1A1 and GAPDH protein levels in cultured HFK cells treated with 59 different proteinases.
3. qPCR analysis of *Col17a1* and *COL17A1* mRNA levels after drug treatment.
4. IF staining for ITGB4 (red), and ZO-1 (green) in DMSO- and Marimastat-treated mice at DPE36. Scale bar = 20 μm.


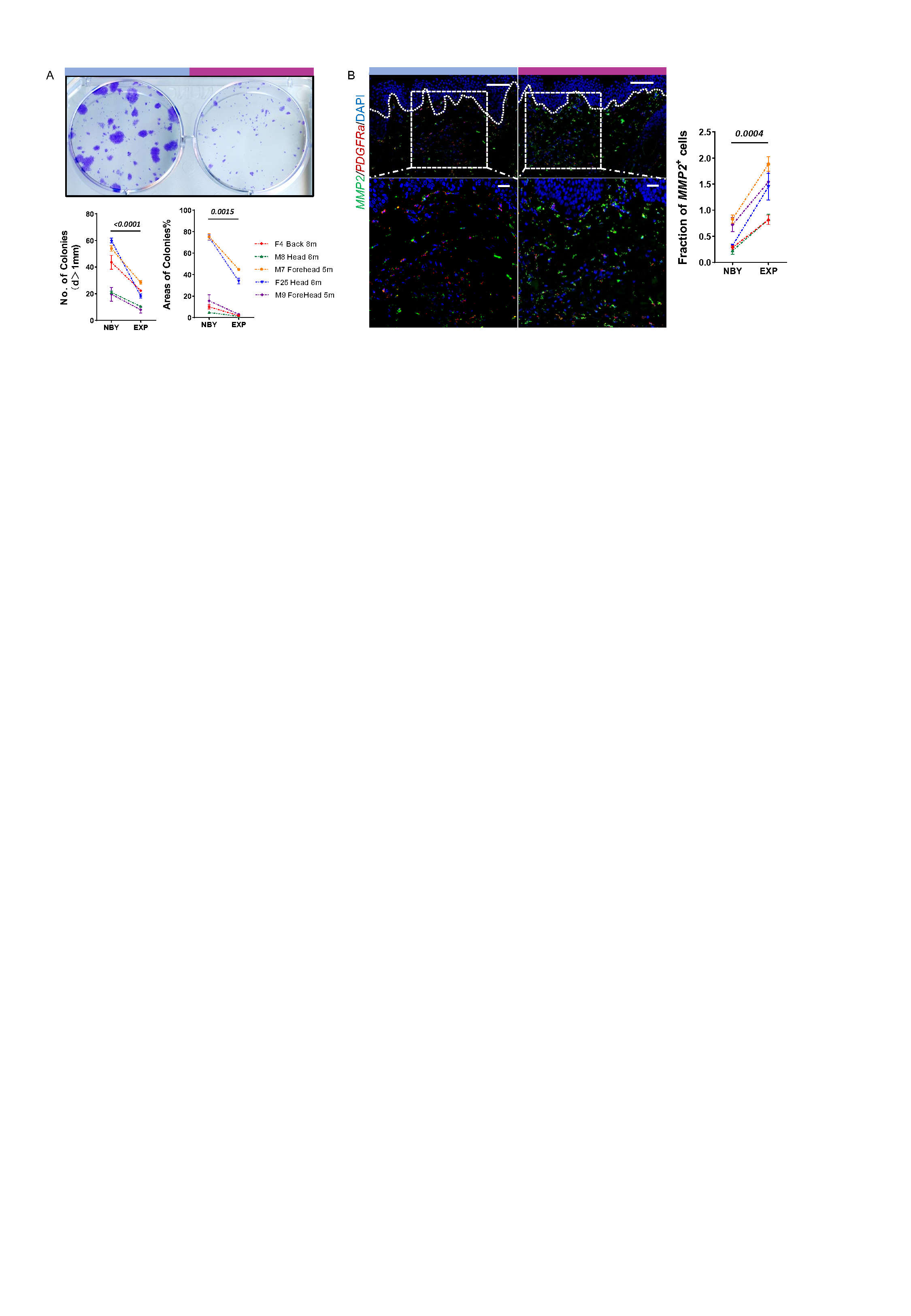


**Figure S7. Human COL17A1 Protein Levels as Indicators of Regenerative Potential in Skin Expansion Therapy**

(A) Representative images of colony-forming assays using epidermal keratinocytes isolated from nearby (NBY) and expanded (EXP) human skin samples. Quantification of the number of large colonies (>4 mm diameter) and total colony area is shown below.

(B) smFISH staining of skin sections from NBY and EXP samples using probes against *MMP2* (green) and *PDGFRα* (red) mRNAs, along with DAPI nuclear counterstaining (blue). Dashed lines delineate the epidermal–dermal boundary. Scale bar = 100 μm. Quantification of *MMP2*⁺ cells in the dermis is shown on the right.

Data represent individual donors, with comparisons analyzed using two-way ANOVA with mixed-effects modeling and Tukey’s post-hoc test.

**Supplementary Methods**

**Histology and immunofluorescence**

Skin samples were harvested immediately after euthanasia and cryopreserved in optimal cutting temperature (OCT) compound (Sakura #4583) for cryosectioning. For immunofluorescence (IF) staining, cryosections were fixed in 4% PFA for 15 minutes and incubated in a blocking buffer containing 2.5% normal donkey serum, 2.5% normal goat serum, 0.3% Triton X-100 and 1% bovine serum albumin (BSA) for 1 hour at room temperature to reduce non-specific antibody binding. Sections were then incubated with primary antibodies at 4 °C overnight, washed with PBS, and further incubated with fluorochrome-conjugated secondary antibodies for 1 hour at room temperature. Slides were mounted using mounting medium with DAPI (Beyotime, P0131). Immunostaining images were acquired using the Olympus FV1200 and Zeiss 880 confocal system and were analyzed with ImageJ software (NIH). Immunohistochemistry (IHC) staining was performed by Servicebio (Wuhan, china). Briefly, IHC samples were prepared by sectioning paraffin-embedded tissues into 5-μm sections, baking at 70°C for 1 hour, deparaffinizing in xylene, and rehydrating through graded alcohol solutions. Antigen retrieval was carried out using citric acid at 95°C for 20 minutes. Sections were treated with 3% H2O2, blocked with 10% goat serum, and incubated with primary antibodies overnight at 4°C. After applying secondary antibodies and streptavidin-peroxidase, slides were washed and developed with DAB, counterstained with hematoxylin, and examined using CaseViewer and ImageJ software. The following antibody dilutions were used: KRT14(chicken, Biolegend, 906004, 1:500), KRT10 (rabbit; Biolegend, 905404, 1:1,000), CD34 (rat; eBiosciences, 14-0341-82, 1:200), KI67 (rat, Invitrogen, 14-5698-82, 1:500; rabbit, abcam, ab15580, 1:500), TRP63 (rabbit, abcam, ab124762, 1:200), Collagen XVII (rabbit, abcam, ab184996, 1:200), ZO-1(mouse, Invitrogen, 61-7300, 1:200 ), ITGA6 (rat, Biolegend, 313602, 1:500), ITGB4 (rat, BD Pharmingen, 553745, 1:500), PLECTIN-1( Rabbit, CST, 12254, 1:200), COL1A1(rabbit, CST, 72026, 1:200), COL3A1 (mouse, Santa Cruz, sc-271249, 1:200), MMP2 (mouse, Invitrogen, 43-6000, 1:200), α-SMA(rabbit, CST, 19245, 1:200), YAP (rabbit, CST, 14074, 1:200), PDGFRA (goat, R&D systems, AF1062, 1:50), F4/80(rat, BioLegend, 123101, 1:400), MMP9 (rabbit, proteintech, 10375-2-AP,1:200). Secondary antibodies used included: Rho-red donkey antibody to rat immunoglobulin (IgG) (Jackson, 712-295-153); Rho-red-conjugated Goat Anti-Mouse IgG (Jackson, 115-295-003); Alexa Fluor® 488-AffiniPure Goat Anti-Chicken IgY (Jackson, 103-545-155); Rho-red donkey antibody to goat IgG (Molecular Probes), Alexa Fluor 647-labeled Goat Anti-Rabbit IgG (Beyotime, A0468), Alexa Fluor® 647-conjugated AffiniPure Donkey Anti-Goat IgG (Jackson, 705-605-147), Alexa Fluor® 488-AffiniPure Goat Anti-mouse IgG (Beyotime, A0428).

Paraffin-embedded sections were stained using a hematoxylin and eosin (H&E) staining kit (Servicebio Tech, G1003) following the manufacturer's protocol to visualize tissue morphology. Frozen sections were stained with Oil Red O (Servicebio Tech, G1016) to identify lipid-rich sebaceous glands. Collagen visualization was achieved through Picrosirius Red staining, with collagen birefringence images captured using a Leica 5000B microscope under orthogonal polarized light.

**Whole skin clarification**

Whole skin samples were harvested from *Krt14-creER;Rosa26-mTmg* mice following expansion and depilation, then fixed in 4% PFA at room temperature for 24 hours. After fixation, the samples were washed with PBS and dissected into uniform pieces measuring 2mm by 2mm for the subsequent clarification process. The CUBIC trial Kit (290-80801, FUJIFILM Wako Puro Chemical Corporation) was employed for whole-skin clarification, adhering to the manufacturer's instructions. Initially, the skin pieces were immersed in a 50% solution of ScaleCUBIC-1 and incubated for 24 hours on a rocking platform set at 100 rpm at room temperature. After this incubation, the samples were transferred to 100% ScaleCUBIC-1 solution and maintained at 37°C for 48 hours in a light-protected environment to ensure thorough clarification. Following this process, the samples were rinsed in PBS for 15 minutes, repeating the wash cycle three times to remove any remaining ScaleCUBIC-1 solution. The procedure was repeated using ScaleCUBIC-2 solution, following the same protocol with both 50% and 100% concentrations. For nuclear visualization, DAPI solution (Beyotime Biotech) was applied, staining the nuclei for 24 hours at room temperature. During imaging, a mixture of 30% mounting solution 1 and 70% mounting solution 2 was used, and the imaging of skin clone cells was performed using a Zeiss microscope equipped with a Z-stack model, which enabled the capture of detailed three-dimensional cellular structures within the clarified skin samples. This approach facilitated high-resolution visualization of cellular morphology and distribution in the expanded skin tissue.

**Cell Culture**

For mouse colony forming efficiency assay ^[1,2]^, freshly expanded skin samples were harvested at specific time points and wiped with alcohol. The stretched skin was then cut, and epidermal sheets were prepared by placing small skin pieces in 0.25% Trypsin-EDTA (GIBICO, 25200056) at 37°C for 2.5 hours. After incubation, the trypsin activity was inactivated using PFE solution (5% FBS, 1 mM EDTA in PBS). The epidermis was gently separated from the dermis with a surgical blade, and mouse keratinocytes (MKs) were dissociated by pipette mixing and filtered through a 40-μm cell strainer. MK viability was assessed using trypan blue staining with an Automatic Cell Counter (HScore, HaloCounter). A total of 4,000 viable MKs were seeded into each well of 6-well plates for colony formation. The cultures were maintained for 12 days post-stretch and for an additional 4 days to reach 16 days for the in vivo drug assay. For human clonal expansion assay, fresh expanded and surrounding nearby skin were obtained and transported in tissue storage solution (Miltenyi, 130-100-008) on ice within 1 hour. After digestion with 5% dispase II (Roche) for 1.5 hours and 0.25% Trypsin-EDTA (Gibico) for 15 minutes, 8,000 viable human primary keratinocytes were seeded into each well of 6-well culture plates and cultured in CNT-Prime growth medium (CNT-PR+1% v/v pen-strep + 10 mM Y-27632) for 18 days. Colonies were fixed, stained with crystal violet (Beyotime #C0121), scanned using an EPSON V500 scanner, and analyzed for clonal number and size using ImageJ software.

For skin explant culture assays ex vivo, according to previous reports^[3,4]^, following hair removal using Veet depilatory cream, expanded skin samples were harvested, and residual cream was washed off with PBS. Subcutaneous fat and fibrous capsule were carefully excised with a scalpel. Explants were generated using a biopsy punch (Kai Medical) to create 1.5 mm diameter discs. These explants were embedded in 1.5 μL of Matrigel (Corning) and placed on culture dishes coated with human plasma fibronectin (Millipore). Keratinocyte cultures were maintained in CNT-Prime Medium (CELLnTECH), and fibroblast cultures were performed in DMEM High Glucose Medium (Gibco). Explant outgrowth was monitored and imaged four days after outgrowth initiation using a Zeiss A1 microscope (Zeiss). The outgrowth area was quantified using ImageJ, and fibroblast numbers were enumerated by Hoechst 33258 staining (Beyotime) and ImageJ analysis. For isolation and culture of human keratinocytes, as previously described, discarded human foreskin samples from plastic surgery wastes were obtained and subjected to overnight digestion with 2mg/ml Dispase II (Roche) at 4°C to isolate the epidermis. The epidermal layer was further digested with 0.25% Trypsin-EDTA (Gibco, 25200056) for 15 minutes at room temperature to release epidermal cells. The dermis was treated with 2.5 mg/mL Collagenase (ThermoFisher) for 45 min at 37°C and cultured in complete DMEM medium. Only early passages of human foreskin keratinocytes (HFKs, passage 3–6) and human foreskin fibroblasts (HFFs, passage 8–10) were used in this study.

For mouse primary keratinocytes culture, backskin from newborn to P4 mice was first digested in 2.5 mg/mL Dispase II (Roche) at 4°C overnight to isolate the epidermis. The epidermis was then digested with Trypsin-Versene (Lonza) for 15 minutes at room temperature to release, wash, and filter the cells. Both human and mouse primary cells were cultured in CNT-Prime growth medium (CNT-PR+1% v/v pen-strep + 10 mM Y-27632) at 37°C in a 5% CO₂ atmosphere.

**Co-culture of HFKs with Feeder Cells and Treatment with Recombinant Human Proteins**

Co-culture of HFKs with mitotically inactivated 3T3-J2 feeder cells was performed as described^[1,5]^. 3T3-J2 cells (HTX2404, Huatuo Bio) were cultured in DMEM with 10% FBS and 1% penicillin-streptomycin. At ~80% confluency, cells were treated with 8 µg/ml mitomycin C for 2h. Concurrently, 6-well plates were coated with 40 µg/ml collagen I (37°C, 2h). Treated 3T3-J2 cells were trypsinized (0.05% Trypsin-EDTA), centrifuged (500×g), and seeded at 8×10⁴ cells/cm² in coated plates overnight. Fibroblast medium was replaced with keratinocyte growth medium (KGM: DMEM/Ham's F12 [3:1] with 10% FBS, 50 IU/ml penicillin-streptomycin, 4 mM glutamine, 0.18 mM adenine, 5 µg/ml insulin, 0.1 nM cholera toxin, 0.4 µg/ml hydrocortisone, 2 nM triiodothyronine, and 10 ng/ml EGF), followed by HFK seeding (10,000 cells/well). Medium was refreshed every 3 days.

For COL17A1 cleavage assays, KGM was supplemented with 50 ng/ml rhMMP2 (TargetMol, A0A024R6R4), rhMMP9 (TargetMol, P14780, used as positive control^[6]^), or BSA (negative control). After treatment, 3T3 cells were removed by gentle pipetting and HFKs were collected for western blot analysis.

**Lentivirus production and infection**

In brief, for the overexpression of human MMP2 (hMMP2), HEK293T cells were transfected with PGMLV vectors carrying hMMP2 cDNA, along with packaging plasmids pMD2.G and psPAX2. Lentiviral particles were harvested 72 hours post-transfection and concentrated via ultracentrifugation at 20,000 rpm for 2 hours at 4°C. The concentrated viruses were subsequently used for HFFs infection.

**Real-time qPCR**

For RNA extraction, cells were first lysed in Trizol^TM^ (Invitrogen, 15596018) or Buffer RZ (Tiangen) reagent. Total RNA was extracted using the RNAsimple Total RNA kit (TIANGEN, DP419) and reverse transcribed with Hifair® III 1st Strand cDNA Synthesis reverse transcription reagent (Yeasen, 11141ES60) according to the manufacturer’s protocol. Complementary DNA was used for real-time PCR with Hieff® qPCR SYBR® Green Master Mix (Yeasen, 11201ES08). Primers are listed in Supplementary Table 2. Relative quantification of gene expression was performed with the comparative threshold method. Changes in mRNA expression levels were calculated after normalization to values for the GAPDH calibrator gene

**Western blots**

For cell protein extraction, RIPA lysis buffer (Beyotime, P0013B) containing proteinase and phosphatase inhibitor cocktail (P1050) was added to culture dishes with cells at 80–90% confluency. Cells were scraped, and the lysate was collected. For tissue protein extraction, mouse skin samples were rinsed in ice-cold PBS, trimmed of excess fat and fascia, cut into 1 mm pieces, and incubated in 2.5 mg/ml Dispase II at 4°C overnight. The following day, the epidermis was separated from the dermis with tweezers, rinsed in cold DPBS, and lysed as previously described. Samples were homogenized using a high-throughput tissue homogenizer and centrifuged at 10,000 rpm for 5 minutes to collect supernatant. Protein concentration was determined using a BCA kit (Epizyme, ZJ101), adjusted to uniform concentration, mixed with 5X SDS loading buffer (Epizyme, L101S), heated at 100°C for 10 minutes, and stored at -80°C. For SDS-PAGE, boiled protein samples were loaded onto a SurePAGE gel (Genscript, M00657) and run at 100 V for 100 minutes. Gels were transferred onto PVDF membranes at 300 mA for 100 minutes under cold conditions, then washed in TBST (3x5 min). Membranes were blocked with blocking solution (Epizyme, PS108) for 30 minutes, washed, and incubated with primary antibodies (Epizyme, PS114) overnight at 4°C. After recovery of primary antibodies, membranes were washed and incubated with secondary antibodies at room temperature for 1–2 hours, developed using ECL solution (Epizyme, SQ201), and visualized. Primary antibodies included Collagen XVII (rabbit, abcam, ab184996, 1:1000), MMP2 (rabbit, abcam, ab97779,1:1000), GAPDH (rabbit, CST, 2118, 1:2000), COL1A1(rabbit, CST, 72026, 1:200), γH2AX (Rabbit, ab81299, Abcam, 1:1000), HISTONE H3 (phospho S10) (rabbit, abcam, ab5176, 1;1000), α-SMA(rabbit, CST, 19245, 1:1000).

**Transmission electron microscopy analysis**

Human and murine skin specimens were sectioned into 1 mm² pieces and fixed in a solution of 2.5% glutaraldehyde and 4% PFA in 0.1 M phosphate buffer (PB) at pH 7.4. Following overnight immersion at 4°C, specimens were washed in 0.1 M PB and post-fixed in 1% osmium tetroxide for 2 hours at 4°C. After rinsing in distilled water, they were stained in 2% uranyl acetate overnight at 4°C. Subsequently, the samples underwent graded ethanol and acetone dehydration, epoxy resin infiltration, and polymerization at 60°C. Ultrathin sections were cut using a Leica UC6 ultramicrotome, mounted on copper grids, and examined with a transmission electron microscope (TEM) equipped with a Gatan 830 CCD camera at the Electron Microscopy Facility (Center for Excellence in Brain Science and Intelligence Technology, Chinese Academy of Science).

For the quantitative assessment of HD width and the density of HDs and micro-delamination, ＞10 TEM images at a magnification of ×50,000 were captured from the basement membrane regions of the interfollicular epidermis for each specimen. Measurements of HDs with or without micro-delaminations, HD width, and basement membrane length were conducted using ImageJ software. The calculated metrics included the number of hemidesmosomes and micro-delaminations per micrometer of basement membrane length. Additionally, intercellular spacing was determined from images taken at a magnification of ×20,000.

**Atomic Force Microscopy (AFM) measurements**

Freshly expanded mouse skin samples collected at DPE8, DPE20, and DPE36 (representing early, mid, and late expansion stages, respectively) along with untreated control samples were embedded in OCT compound without fixation and stored at −80 °C. Prior to sectioning, tissues were snap-frozen and cryosectioned into 40-μm-thick slices using a cryostat. Sections were mounted onto ADM-compatible dishes (Nest, 801001) pre-coated with 0.01% (w/v) poly-L-lysine (Sigma, P4707) at 37℃ for 16h. After firm tissue adherence, each dish was filled with 2 ml of d-PBS containing protease inhibitors (without EDTA; Roche, 11836170001) and kept at 4 °C until AFM measurements, which were performed on the same day to minimize sample degradation.

AFM measurements were conducted using the NanoWizard 4XP atomic force microscope (JPK, Bruker). A silicon nitride cantilever (Bruker NP-O10; nominal spring constant: 0.24 N/m) was equipped with a 10 μm diameter borosilicate glass bead (Novascan Technologies) affixed using epoxy glue (Araldite). The cantilever spring constant was calibrated using the thermal fluctuation method. Stiffness measurements were performed on epi-stromal regions using contact mode force mapping mode at an indentation velocity of 1 μm/s. For each sample, an 8 × 8 grid of force–distance curves were recorded over a 50 × 50 μm² area. However, due to the limited thickness of the epidermis and the relatively large size of the colloidal probe, stiffness measurements in this layer may be influenced by the proximity to the underlying substrate or adjacent dermal tissue. These factors could affect the accuracy of the values obtained. Therefore, stiffness measurements in the epidermal regions should be interpreted with caution.

Elastic properties were calculated using the JPK Data Processing Software, applying the Hertz contact model to derive the Young’s modulus from each force curve. For each sample, 64 force–distance curves were acquired per field of view. The average Young’s modulus was calculated from qualified curves, with measurements obtained from at least three randomly selected fields per biological replicate. Statistical methods used for comparisons between groups are detailed in the corresponding figure legends.

**Fluorescent in situ hybridization (FISH) of human expanded skin samples**

Human skin samples, collected post-expansion, were immediately fixed in 4% paraformaldehyde (PFA) and shipped at -20°C for subsequent analysis. smRNA-FISH was performed on paraffin-embedded sections using the PinpoRNA Multiplex Fluorescent RNA In Situ Hybridization kit (Pinpoease) following the manufacturer’s protocol. Double staining for *PDGFRA* and *MMP2* was achieved through this FISH process. All tissue sections were counterstained with DAPI to visualize nuclei.

**Quantitative analysis of fluorescence intensity measurements, cellular cross-sectional area, and clone size**

To mitigate batch effects, we employed a protocol in which the maximum number of tissue samples were mounted on a single slide and imaged within the same batch. This approach ensured consistency in antibody concentration, incubation times, staining conditions, and imaging parameters. For the quantification of immunofluorescence intensity, we standardized laser intensity and exposure time across samples. The proteins analyzed included COL17A1, ZO-1, ITGA6, ITGB4, PLECTIN-1, COL1A1, COL3A1, MMP2 and MMP9. Fluorescence intensity measurements were performed using ImageJ software, with some images represented in pseudo-color using the Fire mode for visual clarity.

For clone size quantification, whole-mount skin preparations from *Krt14-creER;Rosa26-mTmG* mice underwent skin clarification and imaging using a Zeiss 880 confocal microscope. Basal cells were identified through an orthogonal view that confirmed their direct contact with the dermis and extending hair follicles. This three-dimensional approach enabled accurate counting of GFP-positive basal and total cells per clone. Cells were classified as basal if they were in direct contact with the dermis.

For precise identification of cell contours and quantification of the cellular and nuclear areas of tdTomato+ basal stem cells, we utilized Cellpose software (v2.2.3)^[7]^ in combination with OpenCV's *fillPoly* and *contourArea* contour functions to determine the location and area of the cells. The results allowed us to accurately measure the area occupied by individual cells, converting pixel dimensions into real-world measurements using a calibrated scale bar in each image.

**Construction of scRNA-seq libraries**

Single cells were captured in droplet emulsions and scRNA-seq libraries were constructed according to manufacturer’s protocol using the Chromium 10x Single-Cell Instrument (10x Genomics) and 10x Genomics Chromium Single Cell 3’ GEM Library and Gel Bead Kit v2. Briefly, single cell suspension was resuspended in the master mix and loaded together with partitioning oil and gel beads into the chip to generate the gel bead-in-emulsion (GEM). The poly-A RNA from the cell lysate contained in every single GEM was retrotranscipted to cDNA, which contains an Illumina R1 primer sequence, Unique Molecular Identifier (UMI) and the 10x Barcode. The pooled barcoded cDNA was then cleaned up with Silane DynaBeads, amplified by PCR and the apropiated size fragments were selected with SPRIselect reagent for subsequent library construction. During the library construction Illumina R2 primer sequence, paired-end constructs with P5 and P7 sequences and a sample index were added. All the libraries were sequenced on the NovaSeq 6000 Sequencing System (Illumina).

**RNA velocity-based analysis**

We utilized the scVelo v0.2.5 package^[8]^ for Python ([GitHub repository](https://github.com/theislab/scvelo)) to calculate the ratio of spliced to unspliced mRNA abundances in the dataset. Focusing on the IFE epithelial compartment, data were subclustered and then processed using default parameters in the Scanpy-scVelo implementation. Initial preprocessing involved applying functions for detection of minimum counts, filtering, and normalization using scv.pp.filter_and_normalize, followed by scv.pp.moments with default settings. Next, gene-specific velocities were calculated using scv.tl.velocity with the mode set to stochastic, and velocity graphs were generated with scv.tl.velocity_graph. Finally, we visualized these results with scv.pl.velocity_graph.

**Reference**

[1] K. B. Jensen, R. R. Driskell, F. M. Watt, Assaying proliferation and differentiation capacity of stem cells using disaggregated adult mouse epidermis, *Nat. Protoc.* **2010**, *5*, 898.

[2] F. Liu, X. Zhang, Y. Peng, L. Zhang, Y. Yu, P. Hua, P. Zhu, X. Yan, Y. Li, L. Zhang, miR-24 controls the regenerative competence of hair follicle progenitors by targeting Plk3, *Cell Rep.* **2021**, *35*, 109225.

[3] B. E. Keyes, S. Liu, A. Asare, S. Naik, J. Levorse, L. Polak, C. P. Lu, M. Nikolova, H. A. Pasolli, E. Fuchs, Impaired Epidermal to Dendritic T Cell Signaling Slows Wound Repair in Aged Skin, *Cell* **2016**, *167*, 1323.

[4] Y. Yu, X. Zhang, F. Liu, P. Zhu, L. Zhang, Y. Peng, X. Yan, Y. Li, P. Hua, C. Liu, Q. Li, L. Zhang, A stress-induced miR-31–CLOCK–ERK pathway is a key driver and therapeutic target for skin aging, *Nat. Aging* **2021**, *1*, 795.

[5] E. Enzo, A. Secone Seconetti, M. Forcato, E. Tenedini, M. P. Polito, I. Sala, S. Carulli, R. Contin, C. Peano, E. Tagliafico, S. Bicciato, S. Bondanza, M. De Luca, Single-keratinocyte transcriptomic analyses identify different clonal types and proliferative potential mediated by FOXM1 in human epidermal stem cells, *Nat. Commun.* **2021**, *12*, 2505.

[6] S. Laval, H. Laklai, M. Fanjul, M. Pucelle, H. Laurell, A. Billon-Galés, S. Le Guellec, M.-B. Delisle, A. Sonnenberg, C. Susini, S. Pyronnet, C. Bousquet, Dual roles of hemidesmosomal proteins in the pancreatic epithelium: the phosphoinositide 3-kinase decides, *Oncogene* **2014**, *33*, 1934.

[7] C. Stringer, T. Wang, M. Michaelos, M. Pachitariu, Cellpose: a generalist algorithm for cellular segmentation, *Nat. Methods* **2021**, *18*, 100.

[8] V. Bergen, M. Lange, S. Peidli, F. A. Wolf, F. J. Theis, Generalizing RNA velocity to transient cell states through dynamical modeling, *Nat. Biotechnol.* **2020**, *38*, 1408.
